# Ancient developmental genes underlie evolutionary novelties in walking fish

**DOI:** 10.1016/j.cub.2024.08.042

**Published:** 2024-09-26

**Authors:** Amy L. Herbert, Corey A.H. Allard, Matthew J. McCoy, Julia I. Wucherpfennig, Stephanie P. Krueger, Heidi I. Chen, Allex N. Gourlay, Kohle D. Jackson, Lisa A. Abbo, Scott H. Bennett, Joshua D. Sears, Andrew L. Rhyne, Nicholas W. Bellono, David M. Kingsley

**Affiliations:** 1 Department of Developmental Biology, Stanford University School of Medicine, Stanford, CA 94305, USA; 2 Department of Molecular and Cellular Biology, Harvard University, Cambridge, MA 02138, USA; 3 Department of Pathology, Stanford University School of Medicine, Stanford, CA 94305, USA; 4 Roger Williams University, Bristol, RI 02809, USA; 5 Marine Biological Laboratory, Woods Hole, MA 02543, USA; 6 Howard Hughes Medical Institute Stanford University School of Medicine, Stanford, CA 02543, USA; 7 These authors contributed equally; 8 Lead contact

## Abstract

A critical question in biology is how new traits evolve, but studying this in wild animals remains challenging. Here, we probe the genetic basis of trait gain in sea robin fish, which have evolved specialized leg-like appendages for locomotion and digging along the ocean floor. We use genome sequencing, transcriptional profiling, and interspecific hybrid analysis to explore the molecular and developmental basis of leg formation. We identified the ancient, conserved transcription factor *tbx3a* as a major determinant of sensory leg development. Genome editing confirms that *tbx3a* is required for normal leg formation in sea robins, and for formation of enlarged central nervous system lobes, sensory papillae, and adult digging behavior. Our study establishes sea robins as a model organism for studying the evolution of major trait gain and illustrates how ancient developmental control genes can underlie novel organ formation.

## In brief

Herbert et al. determine that ancient genes involved in limb formation play a key role in the development of new leg-like appendages with unique sensory functions in sea robin fish.

## Graphical abstract

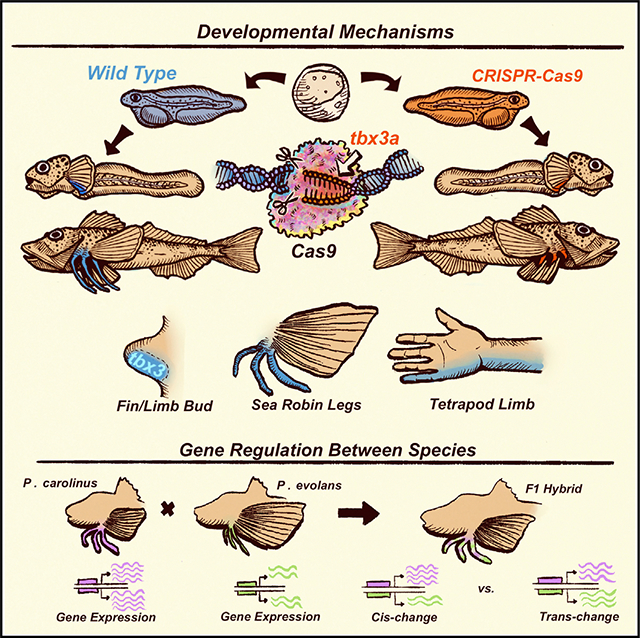

## INTRODUCTION

Understanding the molecular underpinnings of evolutionary change is a key goal in biology. Recent studies have identified multiple examples of loci that control much of the variation in particular traits^1^ including loss of bristles in flies,^2^ loss of pigment in cavefish,^3^ loss of legs in snakes,^4^ and reduction of armor plates and pelvic spines in sticklebacks.^5,6^ Frequently, key evolutionary loci encode essential developmental regulators, with *cis*-regulatory mutations making it possible to avoid deleterious phenotypes and confine major effects to particular tissues.^2,4,6–10^ However, many of the best understood examples of evolutionary change involve trait loss. Whether the evolution of gained traits involves similar types of key developmental genes and *cis*-acting regulatory changes is not yet clear. Moreover, although computational studies have begun to examine the molecular basis of novel vertebrate traits, such as the evolution of flight in birds and mammals,^11^ headgear in ungulates,^12^ and echolocation in bats and cetaceans,^13^ it has usually not been possible to functionally test the hypotheses generated from comparative genomic studies in the same species.

Sea robins are bottom-dwelling marine fish that exhibit multiple striking evolutionary novelties, including enlarged, wing-like pectoral fins, six prominent detached “legs,” with sensory and locomotor capabilities, and the formation of enlarged lobes in the central nervous system (CNS) that allow the animals to walk, dig, and detect food on the ocean floor.^14–16^ Sea robin leg development is a particularly compelling example of trait gain because it involves alterations to multiple tissue types, including skeletal, muscular, and nervous system structures.^17^ Moreover, within the sea robin family there are a small number of sensory specialists that have evolved novel structures that promote digging behaviors, while closely related congenerics do not dig (See Allard et al.^18^ in this issue of *Current Biology*), making sea robins excellent organisms for comparative evolutionary studies.

## RESULTS

### Developmental mechanisms of leg formation

**Figure 1.**
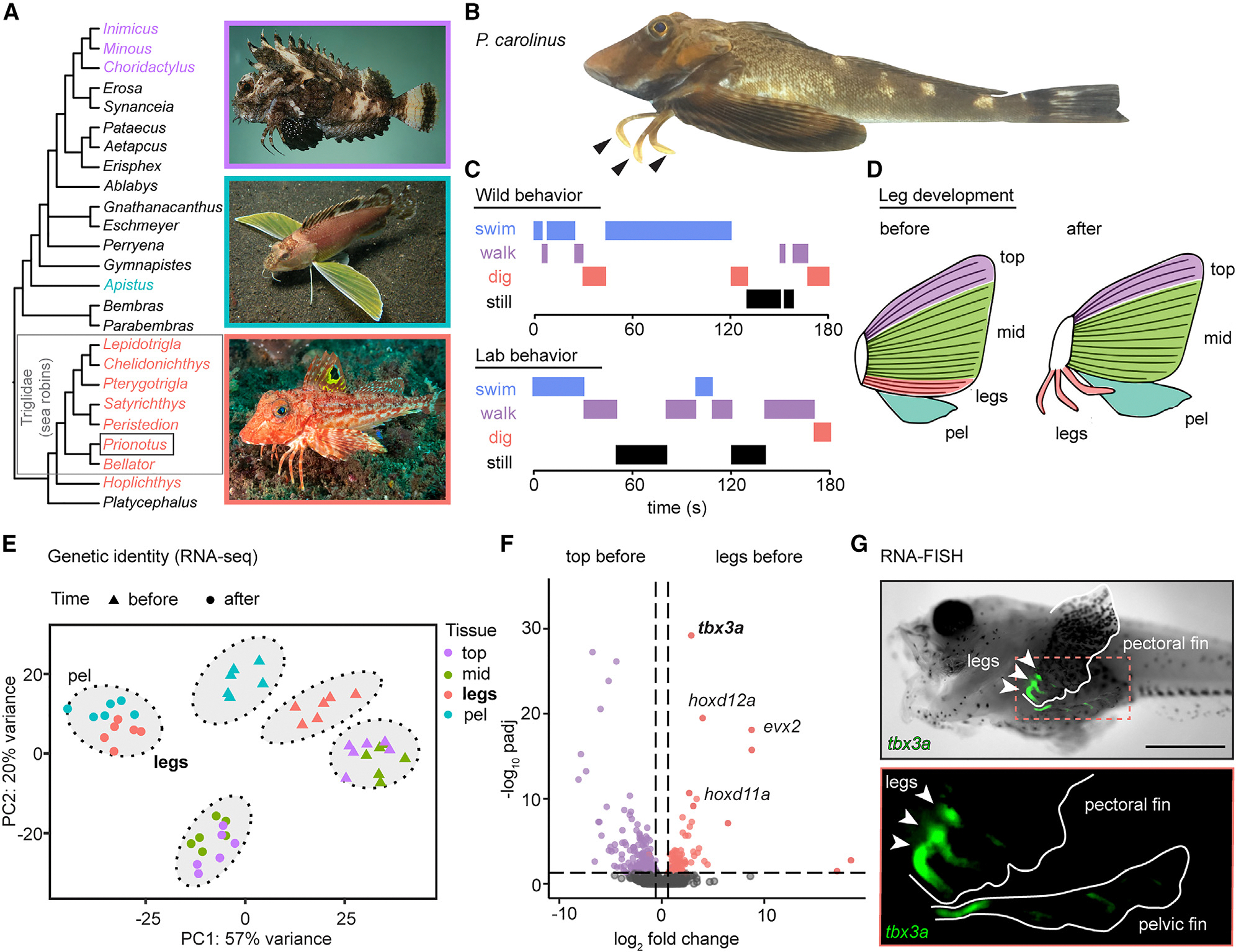
Sea robin leg development and molecular profiling. (A and B) Leg-like structures have evolved at least three separate times, including in the northern sea robin, *Prionotus carolinus*. Tree is based on most recent taxonomic classification of sea robins and relatives.^19^ Photo credits in tree: J.E. Randall, from fishbase.de (top), T. Zuberbühler (middle) and M. Jones (bottom). (C) *P. carolinus* wild animals alternate between swimming, walking, and novel digging behaviors, which were also found in a laboratory setting. (D) Diagrams of fin tissues collected for RNA-seq at two time points, including before and after leg separation. The pectoral fin was dissected into the top three fin rays (top), the middle part of the pectoral fin (mid) and the bottom three rays or legs (legs), depending on the time point. The pelvic fin (pel) was collected in its entirety. (E) A PCA plot with *k*-means clustering of all tissue samples shows that legs are distinct from the other pectoral fin tissues before separation. After separation, legs cluster with pelvic fins instead of the other pectoral fin components. (F) Volcano plot showing upregulated expression of *tbx3a*, *hoxd12a, evx2,* and *hoxd11a* in future legs compared to the top portion of the pectoral fin before leg separation. (G) RNA fluorescent *in situ* hybridization shows *tbx3a* expression in legs before separation. Exact *p*-adjusted values: *padj* = 6.13–30 (F, *tbx3a*), *padj* = 3.23–20 (F, *hoxd12a*), *padj* = 7.86–19 (F, *evx2*), *padj* = 1.05–10 (F, *hoxd11a*). *N* = 6 animals before leg separation and *N* = 6 animals after leg separation. Scale bar, 1 mm (G). See also Figure S1 and Tables S1 and S2.

Sea robins are part of the larger suborder Scorpaenoidei, in which legs appear to have evolved at least three separate times, including in the waspfish (*Apistus*) and in the stingfishes (*Minous, Choridactylus*) and devilfishes (*Inimicus*) (Figure 1A).^19,20^ The northern sea robin *Prionotus carolinus* (*P. carolinus*) is a readily catchable sea robin species in the family Triglidae that exhibits three legs on each side of its body (Figure 1B). Behavioral observations show that wild *P. carolinus* animals alternate between swimming, walking on substrate, and digging in the sand (Figure 1C). Importantly, *P. carolinus* exhibited these same behaviors, including digging, in a natural or laboratory setting (Figure 1C).

To utilize sea robins as an experimental model, we established *in vitro* fertilization and a culturing system to generate embryos and grow larvae for developmental studies (see STAR Methods). Consistent with previous morphological studies,^21,22^ we found that sea robin “legs” develop from the three ventral-most pectoral fin rays, which initially form within the pectoral fin and then separate from the rest of the fin rays during development (Figure S1). Similar to other pectoral rays, legs are composed of two parallel, closely opposed hemitrichia. However, several key skeletal features distinguish adult sea robin legs from pectoral fin rays, including a strikingly robust ventral hemitrichial process (VHP) located at the base of the leg and distinct segmentation patterns along the two leg hemitrichia.^17,23^ Interestingly, movement of legs is not controlled by tendons, but rather actuated by distinct walking muscle groups that insert into the VHP and control movement from the base of the leg.^17,23^

To molecularly characterize leg development, we generated haplotype-resolved genome assemblies of *P. carolinus* using PacBio long-read sequencing and Omni-C sequencing. The high-quality genome assembly used for analysis was approximately 642 Mb in total with an N50 of ~19 Mb and with a complete and single-copy Benchmarking Universal Single-Copy Orthologs (BUSCO) score of 98% (Table S1). We then performed RNA sequencing (RNA-seq) before and after the leg rays separated and compared expression patterns in the ventral leg rays (legs), to those of the developing pectoral and pelvic fins (Figure 1D). Although leg rays originate from the pectoral fin, principal-component analysis (PCA) with *k*-means clustering revealed that they are distinct from the rest of the pectoral fin at early stages and then segregate with the pelvic fin after separation (Figure 1E). Some of the top upregulated genes distinguishing developing leg rays from the rest of the pectoral fin include well known developmental transcription factors that have a role in both pectoral fin development as well as tetrapod limb development, such as *tbx3a*, *hoxd12a*, *hoxd11a*, and *evx2*^24–27^ (Figure 1F). Gene ontology analysis of genes upregulated in leg rays compared to the top portion of the pectoral fin showed enrichment for genes in involved in anatomical structure development, cell surface receptor signaling pathways, and neuron differentiation, consistent with the formation of specialized organs that later support both locomotion and sensory processing (Table S2).

Additionally, we identified markers of conventional pectoral fin rays, including *tbx15* and the actinodin genes *and1* and *and2* (Figure S1). *Tbx15* has a role in shoulder girdle formation and is expressed in zebrafish pectoral rays,^28,29^ whereas *and1*/*and2* encode major structural components of fish fin rays. Interestingly, loss of these two genes in land animals has previously been associated with the evolutionary transformation of fins to limbs in tetrapods.^30^ Reduced expression of actinodin genes in sea robin legs highlights the structural differences between legs and pectoral fin rays in a walking fish lineage. The sequencing studies provide a set of marker genes that distinguish leg and fin fate, as well as candidate genes that sea robins may use to specify their novel locomotory appendages.

One of the top differentially expressed genes between the leg rays and other fin rays in the pectoral fin prior to leg separation was *tbx3a* (Figure 1F) and fluorescent *in-situ* hybridization confirmed strong expression of *tbx3a* in developing sea robin legs (Figure 1G). *Tbx3a* was also one of the top upregulated genes in legs compared to the top pectoral fin (*padj* = 8.97–31) and middle pectoral fin (*padj* = 1.27–21) after separation, suggesting that *tbx3a* may be playing multiple developmental roles. Interestingly, *tbx3a* was also one of the top upregulated genes in the pelvic fin compared to the top pectoral fin. *Tbx3* encodes a T-box transcription factor and is present in a genomic cluster with *tbx5*, a master regulator of forelimb development.^31^ Although *tbx3a* was duplicated in the teleost lineage that includes sea robins, we only observed differential expression of *tbx3a*, not *tbx3b*, in the fin tissues sequenced. Interestingly, humans heterozygous for mutations in *TBX3* have skeletal alterations that primarily affect structures that develop from the posterior region of the forelimb.^32,33^ The expression of *tbx3a* in the leg ray region of the developing sea robin pectoral fin and the regional specificity of the skeletal alterations in humans suggested that *tbx3a* could play a critical role in walking leg ray formation.

### Functional testing of sea robin genes by genome editing

**Figure 2.**
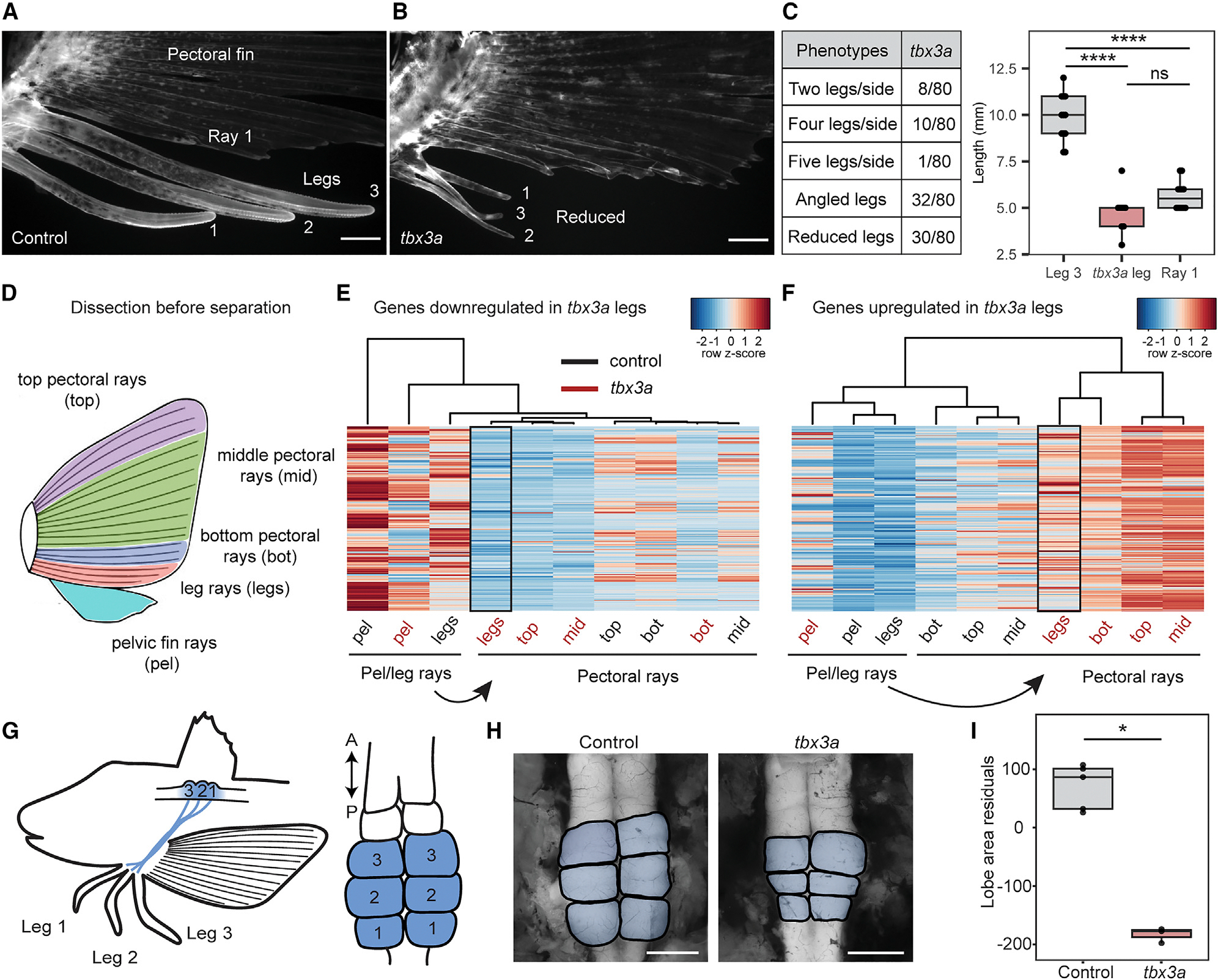
*Tbx3a* disruption alters leg and lobe development. (A) A lateral view of numbered WT legs in a control sea robin. (B) Legs 1, 2, and 3 are reduced in a *tbx3a* crispant, and leg 1 is angled away from its usual position. (C) Quantification of crispant phenotypes in *tbx3a* injected animals (number of animals with phenotype/total number of animals). Length comparison of control leg 3 to *tbx3a* reduced legs and control pectoral ray 1. Significance determined using an ANOVA with Tukey’s HSD post hoc test. *N* = 6 control animals (*n* = 12 legs, *n* = 12 rays) and *N* = 6 *tbx3a* crispants (*n* = 9 reduced legs). Ns = not significant, *p* ≥ 0.05, ****p < 0.0001. (D) Schematic of pectoral fin dissection into the top three pectoral rays (top), the middle pectoral rays (mid), the bottom three pectoral rays (bot) and the bottom three putative leg rays before separation (legs). The entire pelvic fin (pel) was collected. (E) A heatmap of genes downregulated in *tbx3a* crispant legs compared to control legs (*padj* < 0.1). (F) A heatmap of genes upregulated in *tbx3a* crispant legs compared to control legs (*padj* < 0.1). *N* = 8 control animals and *N* = 10 *tbx3a* crispants. (G) Diagram of the 1:1 innervation relationship between sea robin legs and CNS lobes. (H) Representative examples of control and *tbx3a* crispant lobes (lobes pseudo-colored in blue). (I) Lobe area was reduced in *tbx3a* crispants, as measured by regressing against standard length (*N* = 5 control animals and *N* =3 *tbx3a* crispants, *p* = 0.04 by Wilcoxon rank-sum test). Scale bars, 1 mm (A), (B), and (H). See also Figures S2–S5.

To test the functional relevance of the genes identified through RNA-seq studies, we established CRISPR-Cas9 genome editing in sea robins. We first targeted the pigmentation gene *slc24a5*, which provided a visual readout of successful editing^34^ (Figure S2). All injected larvae showed obvious pigmentation abnormalities, and genomic amplicon sequencing showed an average of 81% mutant reads at the *slc24a5* locus, indicating that editing is highly efficient. Genome editing was done in the F0 generation, and the resulting fish (called crispants) were mosaic for multiple mutations.^35^ We next used CRISPR-Cas9 genome editing to target *tbx3a*, one of the top upregulated genes in developing legs identified in our developmental RNA-seq studies. Again, CRISPR-Cas9 was highly efficient (Figure S2). Intriguingly, while disruption of *tbx3a* led to a variety of phenotypes–including angled legs and both decreases and increases in the number of legs from one to five–a notable phenotype was the development of smaller reduced legs, which were visually more similar to pectoral fin rays (Figures 2A–2C). Moreover, we found a significant reduction when we compared the length of the control leg closest to the pectoral fin (leg 3) to the reduced legs from *tbx3a* animals (Figure 2C). Furthermore, the reduced *tbx3a* legs were similar in length to the first pectoral fin ray next to leg 3 (ray 1) (Figure 2C). Examination of skeletal preparations showed that the reduced legs also exhibited fewer bone segments (Figure S3). Additionally, the width of the VHP as well as the total width, which spanned the VHP to the next hemitrich, were both significantly reduced in the *tbx3a* reduced legs compared to leg 3 of the control animals (Figure S3). Thus, multiple distinctive features of sea robin leg morphology fail to form normally after disruption to *tbx3a*.

### Leg and lobe changes in *tbx3a*-targeted fish

The observation that reduced legs in *tbx3*a crispants showed morphological similarities to fin rays led us to hypothesize that *tbx3a* might be a key driver of leg specification. To understand the molecular consequences of *tbx3a* disruption in legs, we performed RNA-seq profiling of *tbx3a* crispants and control siblings before and after leg separation. When we compared the genes downregulated or upregulated in *tbx3a* legs before separation to control legs, we found that legs of *tbx3*a crispants clustered with pectoral fins instead of with the legs of the controls (Figures 2D–2F). Furthermore, we found that 40% of the pectoral fin marker genes were upregulated in *tbx3a* crispant leg rays compared to those of control leg rays (Figure S4), whereas none of these genes were upregulated in control legs compared to *tbx3a* crispants (Figure S4B). Several key genes previously found to be expressed in zebrafish fins during development were found to group *tbx3a* crispant legs together with pectoral fin tissues, including *hpdb*, *bhlha9*, *tbc1d4*, *hsd11b1la*, *slc25a34*, and *hgd*.^36^ Interestingly, we also saw an upregulation of genes involved in pigmentation pathways, including *pmela*, *tyr*, *slc24a5*, and *tyrp1b* (Figure S4C), consistent with a previously studied role for *Tbx3* in pigmentation suppression in horses.^37^ Overall, these results demonstrate that disruption of *tbx3a* results in upregulation of pectoral fin markers prior to leg separation, indicating that leg rays become more similar to fins in the absence of *tbx3a*.

We performed a similar molecular analysis on individual legs from *TBX3* crispants and uninjected controls after leg separation. We again focused on the leg closest to the pectoral fin (leg 3), which is typically the longest leg in WT animals. Approximately 20% of pectoral fin marker genes were upregulated in *tbx3a* crispant leg 3 (Figure S5A). In contrast, only 0.4% of pectoral fin marker genes were upregulated in uninjected leg 3 compared to *tbx3a* crispant leg 3 (Figure S5B). We note that some crispants had two legs instead of three, and one crispant had four legs, an example of the variable phenotypes that result from targeting *tbx3a* in sea robins which resembles both the increases and decreases in digit number previously reported in human patients with Ulnar-mammary syndrome.^33^ Comparison of genes upregulated in pectoral fins and leg 3 highlights the 45 genes driving *tbx3a* crispant leg 3 to become more similar to the pectoral fin, including several fin genes from zebrafish studies (*csf1b*, *ntd5*, *grem1b*, *dlc1*, and *lamb2l*) (Figure S5C;^36^). We also identified expression of specific pectoral fin genes in leg 3 of *tbx3a* crispants, including *actinodin* genes and *tbx15* (Figure S5D). The upregulation of these fin ray genes in *tbx3a* crispant legs supports a key role for *tbx3a* in promoting a leg versus pectoral fin ray fate.

In addition to evolving novel legs from pectoral fins, sea robins also exhibit striking elaborations of the dorsal horn of the spinal cord that are organized in a one-to-one relationship with legs and are thought to play a role in leg function.^16,17^ To further investigate how loss of *tbx3a* might functionally impact sea robins, we examined the dorsal accessory spinal lobes of the sea robin nervous system. We found that the CNS lobes of sea robin *tbx3a* crispant animals were significantly reduced compared to the lobes of control siblings (Figures 2G–2I), demonstrating that disruption to *tbx3a* altered other critical structures involved in leg function. RNA-seq showed low but detectable levels of *tbx3a* expression in adult lobes and spinal cords (Figure S5E). This suggests that the lobe defect could result directly from disruption of neuronal *tbx3a* or could be a secondary effect of aberrant leg development, potentially resulting from a decrease in innervated leg area.

### *Cis*- and *trans*-regulatory control of species-specific differences

**Figure 3.**
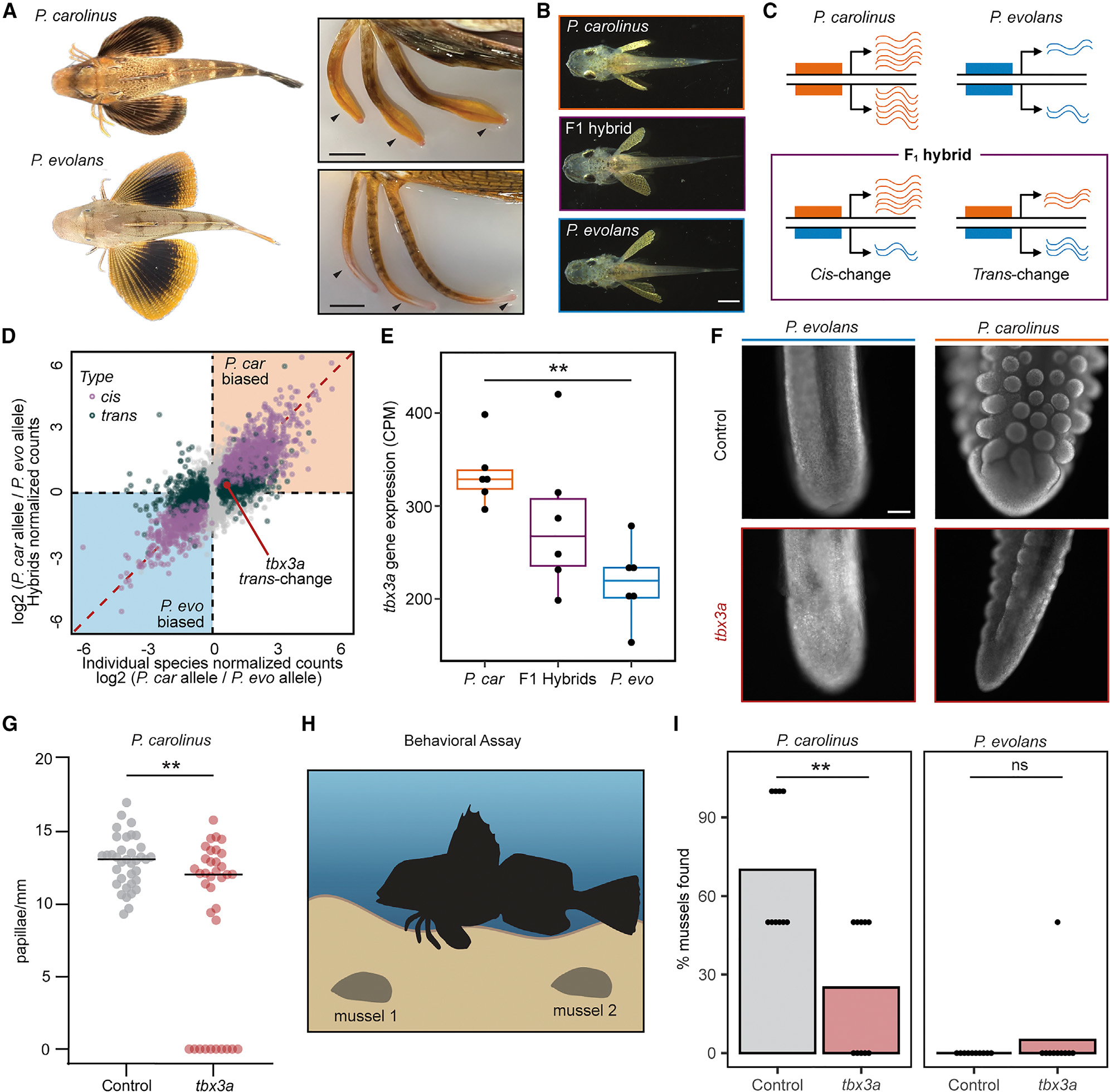
Genetic regulation of species-specific expression changes and sensory structures. (A) *P. carolinus* and *P. evolans* exhibit species-specific differences, including larger wingspan in *P. evolans* and thicker legs with a shovel-like structure in *P. carolinus*. (B) Dorsal views of *P. carolinus*, F1 hybrid, and *P. evolans* animals. (C) A diagram shows how *cis*- and *trans*-regulation can be delineated by comparing gene expression differences seen between two separate species with allele-specific expression differences that are either maintained or lost in F1 hybrid animals. (D) Scatterplot showing gene expression differences between the two species (x axis; *N* = 6 *P. carolinus*, *N* = 5 *P. evolans*) and the differences between the parental alleles in the F1 hybrid animals (y axis; *N* = 6 F1 hybrids) in legs after separation. Genes are labeled as *cis*-regulated (purple; differential gene expression *padj* < 0.05 and allele-specific expression *padj* < 0.05) or *trans*-regulated (dark green; differential gene expression *padj* < 0.05 and allele-specific expression *padj* ≥ 0.05). Identity line is shown in red. (E) Boxplot showing RNA expression of *tbx3a* in counts per million (CPM) in *P. evolans,* F1 hybrids, and *P. carolinus* in legs after separation. Expression is higher in *P. carolinus* compared to *P. evolans* (log2 fold change = 0.67, *padj* = 0.0027). (F) Control *P. carolinus* legs show abundant papillae (*N* = 5/5) that were reduced by *tbx3a* disruption (*N* = 2/3), whereas *P. evolans* legs lacked papillae in both control (*N* = 3/3) and *tbx3a* disrupted conditions (*N* = 3/3). (G) Quantification of papillae density in *P. carolinus* controls (*N* = 6) and *tbx3a* crispants (*N* = 6). A Wilcoxon rank-sum test was used (*p* = 0.0086). (H) Schematic of behavioral assay used to test sensory behavior of sea robins (created with BioRender.com). (I) Digging behavior was reduced in *tbx3a* crispants versus control *P. carolinus* (*p* = 0.0038), closer to levels of non-digging *P. evolan*s controls that were unaffected by *tbx3a* disruption (*p* = 0.368). Ns = not significant, *p* ≥ 0.05. Two mussels were buried per trial. Discovery of both mussels resulted in a score of 100%, while uncovering one mussel was scored as 50%. *N* = 10 trials across 6 animals per genotype used in analysis. A Wilcoxon rank-sum test with BenjaminiHochberg correction for false discovery rate was used. Scale bars, 1 cm (A, both images), 1 mm (B, all three images), and 100 μm (F, all images). See also Figures S6–S10, Tables S1 and S3.

*Prionotus evolans* (*P. evolans*) is another accessible sea robin species inhabiting the same locale as *P. carolinus*. Although both *P. carolinus* and *P. evolans* develop legs from their pectoral fins, the resulting legs show striking morphological and functional differences. We found the wider legs of *P. carolinus* exhibit novel sensory organs involved in gained digging behaviors whereas *P. evolans* exhibits thinner legs that are primarily used for locomotion (Allard et al.^18^; Figures 3A and S6). To further develop this comparative model, we generated haplotype-resolved genome assemblies of *P. evolans* to analyze the genetic basis of leg formation and function. The assembly used in analysis was approximately 819 Mb in length with an N50 of 14 Mb and was of high quality with a complete and single-copy BUSCO score of 94.5% (Table S1). Using *in vitro* fertilization, we successfully crossed *P. evolans* females and *P. carolinus* males to produce viable hybrid animals that survived through the leg-separation stage (Figure 3B). To analyze the molecular basis of diversification of leg function, we collected legs and fins from both individual species and hybrid animals before and after leg separation. We used RNA-seq to identify differentially regulated genes between the two sea robin species, and then tested whether those genes also showed allele-specific expression differences in F1 hybrids.

If a particular difference in gene expression between separate species arose through changes in upstream, *trans*-acting regulatory factors, both alleles of that gene should be expressed at balanced levels in an F1 hybrid, when the two alleles are now present in the same *trans*-acting environment. In contrast, if an interspecific gene expression difference arose because of changes in the *cis*-acting regulatory elements linked to a particular gene, the two alleles of that gene should continue to be expressed at unequal levels in an F1 hybrid^38^ (Figure 3C). Comparisons between the two separate species showed a high percentage of differentially expressed genes, consistent with substantial differences in leg morphology and function (Figure 3D). Many of the genes differentially expressed between separate species continued to show significant allele-specific expression differences when analyzed in the interspecific F1 hybrids, suggesting that numerous leg and fin expression differences arose through changes in *cis*-regulation (adjusted R2 = 0.4553, *p* < 2.2e–16, Figures 3D, S7, and S8). However, many of the genes that were differentially expressed between species showed either balanced expression of the two alleles in F1 hybrids, or partial normalization of allelic expression in hybrids, indicating that *trans*-regulatory changes also make an important contribution to the evolution of sea robin traits.

Gene expression differences were observed in both directions between species. For example, the *aldh1a2* gene, which is involved in retinoic acid synthesis, was expressed at higher levels in the pectoral fins of *P. evolans* than *P. carolinus* (log2 fold change = 2.58*, padj* = 0.00087), and this difference was largely maintained in hybrids (log2 fold change = 2.08, *padj* = 0.0079). *Aldh1a2* (or *raldh2*) has a role in outgrowth of limb appendages, and has been hypothesized to contribute to both expansion of pectoral fins in flying fishes,^39^ and reduction of wings in the flightless emu.^40^ Because *P. evolans* has substantially larger pectoral fins than *P. carolinus*, increased expression of the *P. evolans aldh1a2* allele, both in separate species and in F1 hybrids, suggests that *cis*-regulatory changes to *aldh1a2* may contribute to repeated evolution of forelimb size proportions across multiple fish and bird species.

### *Trans*-regulatory control of *tbx3a* in sensory organ development and function

In contrast, *tbx3a* was expressed at higher levels in the legs of *P. carolinus* than *P. evolans* (Figures 3D and 3E), with analysis in F1 hybrids indicating that the expression difference arises primarily from *trans*-acting changes. To test for possible species-specific roles of *tbx3a*, we also targeted this gene in *P. evolans* using CRISPR-Cas9 genome editing. We found developmental leg phenotypes in *P. evolans* crispants that were similar to those observed in *P. carolinus* (Figure S9), suggesting that *tbx3a* plays a shared role in leg formation in both species. However, *P. carolinus* sea robins also characteristically develop wider, more robust legs than *P. evolans* (Figures 3A and S6), as well as the novel presence of epithelial papillae that mediate increased digging and sensory capabilities (Allard et al.^18^). Interestingly, *Tbx3* is required for normal development of both skeletal and soft tissues in humans,^32,33^ and was recently hypothesized to play a role in the formation of epithelial papillae in the stomachs of ruminants.^41^ Moreover, although there was a significant difference in *tbx3a* expression between the two species, F1 hybrids had an intermediate level of *tbx3a* transcripts compared with *P. carolinus* or *P. evolans* (Figure 3E), and hybrid animals have an intermediate papillae phenotype (Figure S10). When we analyzed papillae on *P. carolinus tbx3a* crispant legs, we found a significant reduction in papillae (Figures 3F, 3G, and S10). Strikingly, many crispant fish exhibited thinner legs that frequently even lacked papillae altogether (Figure 3G). As expected, both *P. evolans* control and *tbx3a* crispant animals did not exhibit papillae (Figure 3F).

To test the functional consequences of *tbx3a* disruption, we analyzed sensory digging behavior. In our companion study, we found that *P. carolinus* uses leg papillae as sense organs to efficiently find and unearth buried food items, whereas *P. evolans* does not localize buried prey (Allard et al.^18^; Figures 3H and 3I). To test if *tbx3a* is required for sensory specialization, we analyzed *P. carolinus tbx3a* crispants and found a significant reduction in their ability to localize and uncover buried mussels, close to levels of the non-digging species *P. evolans,* which appears to use legs primarily for locomotion (Figure 3I). Thus, disruption of *tbx3a* had a profound effect on sea robin behavior, consistent with the altered legs and reduced neural elaboration. Though we cannot distinguish whether papillae changes arise from differences in early leg specification or from a direct role of *tbx3a* in later papillae development, these results show that *tbx3a* is regulated in *trans*- and is critical for leg development, papillae formation, and behavioral function in sea robins.

## DISCUSSION

Walking on legs has evolved multiple times in different organisms, including in sea robins. Here, we show that leg, lobe, and papillae formation in *Prionotus* requires the key developmental transcription factor *tbx3*a. *Tbx3* has high expression and a role in posterior limb development in many vertebrates, including humans, mice, chickens, and other fish species that do not form walking leg rays.^26,27^ This ancient early expression pattern likely reflects a conserved role in patterning structures within the postaxial zone of the forelimb bud. Notably, humans with Ulnar-mammary syndrome show changes in an anatomically specific set of skeletal structures that form within this postaxial zone (the ulna bone but not the radius bone in the forearm, and posterior but not anterior digits in the hand). Similarly, evolutionary specializations in sea robins occur in the postaxial region of the pectoral fin, with three fin rays in this zone detaching from the rest of the pectoral fin and developing into robust walking and sensory rays. Although digits in tetrapods and fin rays in fish are not homologous structures, they do share developmental histories.^9^ Our studies illustrate how an ancient patterning gene can be involved in both posterior region-specific skeletal defects in humans and posterior region-specific evolutionary specializations in a walking fish lineage.

Although sea robin legs are derived from pectoral fins, our transcriptional profiling shows interesting similarities between leg appendages and pelvic fins, particularly after separation. We believe these similarities may partially reflect the development of thicker and more robust skeletal structures in fin derivatives that support body weight on the ocean floor. Many benthic-dwelling fish species, including sculpins and sea robins, rest upon their pelvic fins when in contact with the ocean floor. Genes with greater than a 10-fold expression difference between weight-supporting fin derivatives (legs and pelvic fins) versus flexible swimming structures (top pectoral fin rays) include *hyaluronan synthase 1* (*has1*), which is known to control size and strength of skeletal structures in other animals.^42^

In both humans and sea robins, mutations in *TBX3* also lead to notable variability in limb phenotypes. For sea robin crispants, this variability could arise in part from the range of mutations and degree of mosaicism following genome editing. We note, however, that human families show similar variability in skeletal phenotypes even when inheriting an identical heterozygous *TBX3* mutation in every cell of the body.^33^ An important future goal will be to transmit individual sea robin *tbx3a* mutations through the germ line and examine the range of fin and sensory leg phenotypes seen in walking fish that are heterozygous or homozygous for *tbx3a* mutations throughout the body.

A striking finding in the *tbx3a* crispant animals was the reduction of the spinal lobes. In zebrafish, *tbx3a* is expressed in several neuronal cell types during development, including motor neurons and retinal ganglion cells.^36,43,44^ Our transcriptomic analysis identified low but detectable levels of *tbx3a* in adult lobes and spinal cords, and it is possible that *tbx3a* also functions in developing lobes at earlier stages. Alternatively, altered development of the peripheral appendages of *tbx3a* crispants may lead to indirect changes in the size of central nervous system structures innervating the legs. Future experiments will examine the ontogenetic relationship between legs and lobes to determine if lobe reduction results from disruption to leg development and function, or whether *tbx3a* has an additional autonomous role in lobe development.

How could the novel evolutionary structures of sea robins arise through an ancient transcription factor that is expressed in a conserved postaxial zone in vertebrate appendage development? One possibility is that early postaxial expression of *tbx3a* is prolonged in the sea robin lineage relative to other legless fish species, allowing formation of late-developing specializations in walking fish. Future studies will assay expression of *tbx3a* at later stages in legless relatives and look for potential upstream *cis*- or *trans*-regulatory changes that could lead to alterations in levels or timing of postaxial expression. Another possibility is that the *tbx3a* expression domain is conserved, but sea robin-specific changes in the amino acid sequence of *tbx3a* lead to new downstream gene expression programs within this ancient expression domain. The sea robin *tbx3a* protein sequence does show multiple derived amino acid changes present in both *P. carolinus* and *P. evolans* that are not present in legless outgroup species, including sticklebacks, medaka, and zebrafish (Table S3). Because genome editing is now possible in both sea robins and other legless fish, it should be possible to test the importance of species-specific amino acid changes by reverting or recreating specific amino acid changes within the *tbx3a* gene and testing for effects on fin and leg ray development. Finally, sea robins may have evolved novel target genes responding to the ancient *tbx3a* transcription factor, perhaps by gaining new binding sites in a variety of downstream genes involved in leg ray skeletal or sensory functions. Our gene expression studies identify hundreds of genes whose expression in sea robins is particularly sensitive to *tbx3a* disruption. Future studies can examine whether some of these genes have evolved sea robin-specific *tbx3a* binding sequences that have recruited the genes into new roles in sensory leg formation.

The methods established here provide a new model for combining genomic and functional studies of evolutionary innovations within the larger group of sea robin relatives, which encompass a huge diversity of phenotypes, including changes to pectoral and pelvic fins, evolution of new lobes in the nervous system, development of armor and venom, sound production, and evolution of dramatic coloration patterns. Although functional genomic studies are difficult in many vertebrates, the novel traits, large egg clutches, and ability to culture, hybridize, and edit sea robins provides a powerful system for studying how morphological and behavioral innovations have evolved in wild species.

## RESOURCE AVAILABILITY

### Lead contact

Further information and requests for resources and reagents should be directed to and will be fulfilled by the lead contact, David Kingsley (kingsley@stanford.edu).

### Materials availability

This study did not generate new unique reagents.

### Data and code availability

- All raw sequencing data generated from this study have been deposited at the NCBI Sequence Read Archive and is publicly available as of the date of publication. Accession numbers are listed in the key resources table.
- All original code has been deposited at Figshare and is publicly available as of the date of publication. The DOI is listed in the key resources table.
- Any additional information required to reanalyze the data reported in this paper is available from the lead contact upon request.

## STAR★METHODS

### EXPERIMENTAL MODEL AND STUDY PARTICIPANT DETAILS

#### Ethical compliance and animal care

All relevant ethical regulations were followed during this study. All sea robin studies were performed according to recommendations in the Guide for the Care and Use of Laboratory Animals of the National Institutes of Health.^51^ Protocols for sea robin care and use were approved by the Institutional Animal Care and Use Committees (IACUCs) at the Marine Biological Laboratory (protocol numbers 17–36, 18–08C, 19–28, 20–22, 21–22, and 22–21), Roger Williams University (protocol numbers R19–07-06 and R-22–11–30), Stanford University (protocol number 32297), and Harvard University (protocol number 18–05-324–1).

#### Adult sea robin care and *in vitro* fertilization

Adult *Prionotus evolans* and *Prionotus carolinus* were provided by the Marine Biological Laboratory (MBL) during the reproductive months of May and June. At the MBL, animals were kept in large tanks with constant ambient sea water flow and aeration by a large airstone. Animals were fed every other day. Embryos were obtained through strip spawning. In brief, eggs were obtained through manual expression of reproductive females into a large Petri dish. Milt was obtained through manual expression of reproductive males. Milt was captured by a plastic transfer pipet, mixed with eggs, and allowed to sit for 10 min. 1 μm filtered sea water was then added to developing embryos.

#### Sea robin larval rearing

Sea robin larvae were raised at 18°C in deep well dish petri dishes for three days in 0.22 μm filtered sea water with 130 μL methylene blue/L. Half water changes were done daily. Prior to the hatching, embryos were transported from MBL to Roger Williams University (RWU) in 50 mL conical tubes. At RWU, they were placed in recirculating Modular Larval Rearing Systems (MoLaRS).^52^ To optimize hatching conditions, a salinity of 36 ppt was maintained, with the ambient water temperature consistently between 20°C and 22°C. Larvae hatched on day 4 as yolk sac larvae and initiated first feeding on day five. MoLaRS were pre-stocked with *Parvocalanus* sp. copepods (nauplii) after hatching. As larvae developed, salinity was gradually lowered to lab ambient (30 ppt) and maintained between 30 and 32 ppt during the course of larval development. *Tisochrysis galbana* supplemented with *Tetraselmis* sp. and/or *Nannochloropis* sp. served to provide “greenwater” background contrast, enhanced feeding response, and provided food for copepods. The copepod stocking density was maintained at 2 to 4 per mL for both nauplii and adults. 10–15 days post-hatch, larvae were supplemented with *Pseudodiaptomus pelagicus*. Larvae began setting after approximately 3 weeks and juveniles were fed enriched *Artemia salina* meta-nauplii. Instar 2 *Artemia* were enriched with LARVIVA Multigain (BioMar SAS) at concentration of 0.5 g per 1 L of seawater for 4 h and cold stored (4°C) until feeding. Five weeks post-fertilization, juveniles were transitioned onto B2 TDO Chroma Boost pellets (Reef Nutrition) and fed larger pellets as they grew. E-series sand (Holliston Sand Co Inc, Burrillville, Rhode Island, USA) was introduced to the tanks to prevent potential fin abrasion.

Sex genotyping is not yet possible in sea robins, and therefore sex could not be used as a variable in the developmental studies.

### METHOD DETAILS

#### Sea robin behavior

Adult sea robin lab behavior in the lab was observed as described in Allard et al.^18^ In brief, sea robins were placed in a 1.5 m diameter pool containing 200 gallons of sea water and ~8 cm of sand. A GoPro HERO 7 cameras (GoPro, Inc.) was used to film behavior and footage was evaluated using Windows Media Player (Microsoft). Sea robin wild behavior was filmed by the company Marine Imaging Technologies. Underwater videos were shot near the Marine Biological Laboratory in Woods Hole, MA and evaluated using Windows Media Player (Microsoft).

#### Genome generation

Liver tissue from a female *Prionotus carolinus* and a female *Prionotus evolans* was dissected, flash frozen, and sent to Dovetail genomics for genome generation and assembly. DNA was extracted using a Qiagen Genomic DNA extraction kit. DNA samples were quantified using a Qubit 2.0 Fluorometer (Life Technologies, Carlsbad, CA, USA). The PacBio SMRTbell library (~20kb) for PacBio Sequel was constructed using SMRTbell Express Template Prep Kit 2.0 (PacBio, Menlo Park, CA, USA) using the manufacturer recommended protocol. The library was bound to polymerase using the Sequel II Binding Kit 2.0 (PacBio) and loaded onto PacBio Sequel II. Sequencing was performed on PacBio Sequel II 8M SMRT cells. PacBio CCS reads were used as an input to Hifiasm1 v0.15.4-r347 with default parameters. Blast results of the Hifiasm output assembly (hifiasm.p_ctg.fa) against the nt database were used as input for blobtools2 v1.1.1 and scaffolds identified as possible contamination were removed from the assembly (filtered.asm.cns.fa). Finally, purge_dups3 v1.2.5 was used to remove haplotigs and contig overlaps (purged.fa).

For each Dovetail Omni-C library, chromatin was fixed in place with formaldehyde in the nucleus. Fixed chromatin was digested with DNase I and then extracted, chromatin ends were repaired and ligated to a biotinylated bridge adapter followed by proximity ligation of adapter containing ends. After proximity ligation, crosslinks were reversed and the DNA purified. Purified DNA was treated to remove biotin that was not internal to ligated fragments. Sequencing libraries were generated using NEBNext Ultra enzymes and Illumina-compatible adapters. Biotin-containing fragments were isolated using streptavidin beads before PCR enrichment of each library. The library was sequenced on an Illumina HiSeqX platform to produce ~30x sequence coverage.

The input *de novo* assembly and Dovetail OmniC library reads were used as input data for HiRise, a software pipeline designed specifically for using proximity ligation data to scaffold genome assemblies.^53^ Dovetail OmniC library sequences were aligned to the draft input assembly using bwa (https://github.com/lh3/bwa). The separations of Dovetail OmniC read pairs mapped within draft scaffolds were analyzed by HiRise to produce a likelihood model for genomic distance between read pairs, and the model was used to identify and break putative misjoins, to score prospective joins, and make joins above a threshold. For both genomes, the BUSCO version used was 4.0.5 and the lineage dataset was eukaryote_odb10.^54^

#### Genome annotation

Liftoff^45^ was used to lift over annotations from the haploid *Gasterosteus aculeatus* genome (NCBI RefSeq Assembly GCF_016920845.1) to haploid *P. carolinus* and *P. evolans* genomes using minimap2 parameters -mm2_options = “-r 2k -z 5000”.

#### *In situ* hybridization

RNA *in-situ* hybridization (ISH) was performed to evaluate the expression of *tbx3a* using RNAscope probes and detection reagents (RNAscope Multiplex Fluorescent detection kit v2, CAT NO: 323110; Advanced Cell Diagnostics, Newark, CA). Briefly, sea robin whole-mount larvae were pretreated with RNAscope Hydrogen Peroxide, Protease Plus and Target Retrieval reagents prior to hybridization with the RNAscope Pcar-tbx3a-O1 mRNA oligo probes, or with probes against the bacterial gene *dihydrodipicolinate reductase* (catalog #320758) from *Bacillus subtilis* as a negative control. After 2 h of probe hybridization, preamplifier, amplifier, and HRP-labeled oligos were hybridized sequentially, and detected with vivid dye fluorophores (ACDs). *N* = 4 larval fish were used for *tbx3a* and *N* = 2 fish were used as negative controls.

#### CRISPR-Cas9 genome editing

Single guide RNAs (sgRNAs) were designed in Geneious prime software using the Find CRISPR Sites tool. sgRNAs were generated using previously described methods.^34^ The CRISPR-Cas9 mixture consisted of a final concentration of 300 ng/μL sgRNA(s), 1 μg/ul Cas9–2NLS protein (QB3 MacroLab, University of California - Berkeley), 0.05% phenol red, and 70 kDA fluorescein isothiocyanate-dextran diluted 1:5 in the final mixture. 2 M KCl and water were added to a final concentration of 300 mM KCl.

CRISPR-Cas9 reagents were introduced into sea robin embryos using a microinjection approach at the 1–4 cell stage. In brief, quartz needles (Sutter Instrument, QF100–70-10) were pulled using a P2000 micropipette puller (Sutter Instrument) with the following settings: heat = 600, fil = 4, vel = 60, del = 140, pul = 175. Quartz needles were beveled using a BV10 beveler at 20° for 30 s. Embryos were lined up in a 1% low melt agarose mold and injected using a Sutter XenoWorks Digital injector.

#### Crispant genotyping

Tissues were preserved for genotyping either by fixation in 70% EtOH, or by flash freezing. DNA was extracted using either a DNeasy blood and tissue kit (Qiagen, 69506) or an Allprep DNA/RNA micro kit (Qiagen, 80284). 2x Phusion Green Hot Start II High-Fidelity PCR Master Mix (ThermoFisher Scientific, F566) was used to amplify sequences targeted by the sgRNAs. Products were PCR purified using a QIAquick PCR purification kit (Qiagen, 28106) and quantified using a Qubit DNA High Sensitivity kit (Invitrogen, Q32854). Samples were sent for Amplicon-EZ sequencing at Azenta Life Sciences (2×250bp). The raw Illumina reads were checked for adapters and quality via FastQC. The raw Illumina sequence reads were trimmed of their adapters and nucleotides with poor quality using Trimmomatic v. 0.36. Paired sequence reads were then merged to form a single sequence if the forward and reverse reads were able to overlap. The merged reads were aligned to the reference sequence and variant detection was performed using AZENTA proprietary Amplicon-EZ program. The genotyping results were as follows: 81% percent average mutant reads (*slc24a5* crispants, *N* = 20) and 1.58% mutant reads (controls, *N* = 20). 86% average mutant reads (*P. carolinus tbx3a* crispants, *N* = 21) and 0.5% average mutant reads (controls, *N* = 15). 82% average mutant reads (*P. evolans tbx3a* crispants, *N* = 7) and 1% average mutant reads (controls, *N* = 6).

#### RNA-sequencing and analysis

Juvenile fish were euthanized by immersion in Tricaine-S (Syndel) until 10 min past cessation of opercular movements. Tissues were dissected using microscissors and stored in either RNAlater (Invitrogen, AM7020) or flash frozen. Tissues dissected into RNAlater were stored overnight at 4°C and then stored at −20°C until extraction. Flash frozen tissues were dissected into 1x PBS, liquid was aspirated, and samples were snap frozen in liquid nitrogen and stored at −80°C until extraction. Libraries were prepared either in house or by Azenta Life sciences. For in-house preparations, RNA extraction was performed using either an RNeasy micro kit (Qiagen, 74034) or an Allprep RNA/DNA extraction kit (Qiagen, 80284). RNA concentration was measured by a Qubit RNA High Sensitivity Kit (Invitrogen, Q32855) and RNA integrity (RIN) was checked by Bioanalyzer at the Stanford Protein and Nucleic Acid Facility (if feasible given low input concentrations). RNA input was normalized across samples and RNA libraries were prepared utilizing the SMART-Seq v4 Ultra Low Input RNA Kit for Sequencing (Takara, 634890, 634888) followed by the Nextera XT DNA library preparation kit (Illumina, FC-131–1024, FC-131–1096) per manufacturer instructions. Samples were randomized to prevent batch effects. Sequencing was performed by Novogene on either a HiSeq 4000 or a Novoseq 6000 lane (2×150 bp).

For samples prepared by Azenta, frozen tissues were homogenized using a motorized handheld homogenizor and plastic pestles in low volumes of TRIzol and flash frozen in liquid nitrogen. Randomization was performed to prevent batch effects. RNA extraction, sample QC, library preparations, sequencing reactions, and initial bioinformatic analysis were conducted at GENEWIZ/Azenta Life Sciences LLC (South Plainfield, NJ, USA). Total RNA was extracted from cells using the Qiagen RNeasy Plus Universal Micro Kit following manufacturer’s instructions (Qiagen). Extracted RNA samples were quantified using a Qubit 2.0 Fluorometer (Life Technologies) and RNA integrity was checked using Agilent TapeStation 4200 (Agilent Technologies).

The SMARTSeq HT Ultra Low Input Kit was used for full-length cDNA synthesis and amplification (Clontech), and Illumina Nextera XT library was used for sequencing library preparation. Briefly, cDNA was fragmented, and an adaptor was added using Transposase, followed by limited-cycle PCR to enrich and add index to the cDNA fragments. Sequencing libraries were validated using the Agilent TapeStation and quantified by using Qubit Fluorometer as well as by quantitative PCR (KAPA Biosystems). The sequencing libraries were multiplexed and clustered on a flowcell. After clustering, the flowcell was loaded on the Illumina NovaSeq 6000 instrument according to manufacturer’s instructions. The samples were sequenced using a 2×150 Paired End (PE) configuration. Image analysis and base calling were conducted by the HiSeq Control Software (HCS). Raw sequence data (.bcl files) generated from Illumina HiSeq was converted into fastq files and de-multiplexed using Illumina’s bcl2fastq 2.17 Software. One mismatch was allowed for index sequence identification.

For all experiments, RNA-seq analysis was performed in-house. Raw data quality was assessed using FastQC, version 0.11.9.^55^ Trimming was performed using Cutadapt version 1.18.^46^ Salmon version 1.10.3 was used for calculating transcripts per million for the lobe RNA-sequencing experiment.^50^ *N* = 4 adult animals were used. For performing differential expression analysis, reads were aligned to the annotated *P. carolinus* genome using STAR version 2.7.10b.^47^ Read counts per gene were determined using the parameter –GeneCounts in STAR. Differential expression analysis was performed using DESeq2^48^ in R version 4.2.2. *N* = 6 animals before leg separation and *N* = 6 animals after leg separation were used in the initial RNA-seq experiment (Figure 1). Covariates used to eliminate batch effects in DESeq2 analysis included RNA extraction and library preparation groups. *N* = 8 control animals and *N* = 10 *tbx3a* crispants were used in the *P. carolinus tbx3a* crispant before leg separation experiment (Figure 3). TRIzol homogenization group was used as a covariate. *N* = 4 samples (two *tbx3a* leg samples, one uninjected leg sample, and one uninjected bot sample) were removed due to high sequence duplication levels (visualized in FastQC). *N* = 6 control animals and *N* = 8 *tbx3a* crispants were used in the *P. carolinus tbx3a* crispant after leg separation experiment. *N* = 1 pectoral fin sample from the *tbx3a* crispants was removed before sequencing due to a low RIN score. RNA extraction group was used as a covariate.

For PCA analysis, samples were clustered into groups based on similarity of gene expression profiles using *k*-means clustering. To identify the optimal number of clusters to use for *k*-means clustering, we used the elbow method^56^ where the first two principle components were used to calculate the within-cluster sum of squares for different numbers of clusters. We also applied the silhouette method,^57^ which measures how similar an object is to its own cluster compared to other clusters using Euclidean distances. Both methods agreed on 5 clusters as the optimal number.

Gene ontology analysis was performed using the Gene Ontology Resource site (https://geneontology.org/). Genes upregulated with a padj < 0.1 in legs compared to the top portion of the pectoral fin were used with the biological process database from medaka fish (*Oryzias latipes*). A background gene list from the RNA-seq experiment was also utilized, where only genes with detectable expression were included. A Fisher’s exact test with a false discovery rate < 0.05 was utilized to determine the significance of enrichment.

#### Allele-specific expression analysis

Sequencing quality was confirmed using FastQC, version 0.11.9^55^ and reads were trimmed using Cutadapt version 1.18.^46^ Trimmed reads were aligned to a composite *P. carolinus - P. evolans* genome using STAR 2.7.9a.^47^ Differential gene expression (DGE) between individual species and allele-specific expression (ASE) within hybrids was determined as described previously.^58^ For each tissue and stage (e.g., “legs after”), genes with fewer than 10 reads assigned to the *P. carolinus* and *P. evolans* orthologs in each sample were excluded, and each sample was library-size normalized. Genes with significantly different log2 fold-change between *P. carolinus* and *P. evolans* were determined to have differential gene expression (DGE) or allele-specific expression (ASE) (Welch’s t test; Benjamini-Hochberg correction for false discovery rate, *padj* < 0.05). Genes were considered to be regulated in *cis*- if the log2 fold-change for both DGE and ASE were significant and in the same direction, and *trans*- if significant only for DGE. Principal component analysis was performed after initial processing with DESeq2^48^ using default parameters. In hybrid samples, size factors were estimated before segregating *P. carolinus* and *P. evolans* alleles.

#### Phenotyping of crispants

Phenotyping of *slc24a5* crispants was performed in the Genome Editing Core at the MBL. Reduction of pigment was scored qualitatively (WT, phenotype) on a Zeiss Discovery V20. Images were taken using Axiovision software. *Tbx3a* crispants were phenotyped at RWU and from images of animals taken at RWU. Caudal tails were dissected and placed in 70% EtOH for future genotyping. Length measurements of control leg 3 and *tbx3a* reduced crispant legs were taken on a Zeiss AxioZoom V16 microscope and measured using FIJI.^49^ The length of the first pectoral fin ray and the standard length of fish were measured with a ruler. There was no significant difference in standard length between the two groups (*p* = 0.912, two-sample t test) and this measurement was therefore not used in the analysis. *N* = 6 control animals (*n* = 12 legs and 12 pectoral rays) and *N* = 6 *tbx3a* crispants (*n* = 9 legs) were measured, and an ANOVA with Tukey’s HSD post hoc test was used to assay significance.

#### Lobe measurements and analysis

*Tbx3a* crispants (*N* = 4) and control uninjected siblings (*N* = 5) were euthanized and dissected to expose the dorsal accessory lobes. Standard length of all samples was measured. In FIJI,^49^ the freeform tool was used to measure lobe area. Lobe images were scored blinded to genotype. A non-linear regression model in R version 4.2.2 was used to calculate the residuals of the area measurements taking into account the standard length of the fish. Presence of a leg phenotype in the *tbx3a* crispant individuals was observed from images, and lobe measurements of crispants were pooled from the same sides of the body that showed leg phenotypes (*N* = 2 crispants had unilateral leg phenotypes, *N* = 1 had bilateral leg phenotypes, and *N* = 1 crispant had no leg phenotypes and was excluded). Lobe measurement residuals for samples were averaged and a Wilcoxon rank-sum test was used to determine significance of the differences between controls and *tbx3a* crispants.

#### Pectoral fin and leg measurements

Adult *P. carolinus* and *P. evolans* were anesthetized using clove oil. For pectoral fin measurements, a flexible tape measure was used to measure the standard length (SL) of the fish in addition to the length of the left and right pectoral fins. The middle of the pectoral fins from *N* = 10 *P carolinus* and *N* = 11 *P evolans* were measured from the base of the fin to the tip. The pectoral fin length was adjusted according to the standard length of the fish using a linear regression model in R version 4.2.2 and significance of residuals was determined using a Wilcoxon rank-sum test with Benjamini-Hochberg correction for false discovery rate, exact = FALSE. For leg measurements, animals were again sedated in clove oil and a flexible tape measure was used to measure the standard length. The leg width of all three legs on the left and right of *N* = 13 *P carolinus* and *N* = 13 *P evolans* was measured. Leg width was measured at the leg bend, above the shovel structure. Width measurements were regressed against the standard length of the fish in R. A Wilcoxon rank-sum test with Benjamini-Hochberg correction for false discovery rate was used to determine significance, exact = FALSE.

#### DAPI staining

Whole fish were anesthetized in an overdose of MS-222 and fixed in 4% paraformaldehyde. Tissues (individual legs and/or pectoral fin) were dissected in 1X phosphate buffered saline (PBS) and incubated in solution of 0.5 mg/mL DAPI in PBS for 30–60 min in the dark at room temperature with gentle agitation. Tissues were then washed four times in 1X PBS and imaged in 35 mm polystyrene dishes on a AxioZoom v16 microscope (Zeiss). Image analysis was done in FIJI 2. Data were visualized using R version 4.2.2 and Graphpad Prism.

#### Behavioral analysis

Four groups of six animals (*tbx3a* crispants and wildtype controls, of *P. carolinus* and *P. evolans*) were raised in seawater tanks to ~2 months. Digging behavior experiments were performed using a behavioral model first tested with adult sea robins. Prior to behavioral experiments, tank sand was cleaned via gravel siphoning and animals were not fed directly before.

Blue mussels (*Mytilus edulis*) were purchased from the local market and dissected into 1–1.5 cm pieces for shallow sand burial. Two PVC-pipe pieces were used to shield visual cues while two mussel pieces were separately buried in the sand. The PVC-pipes were removed, allowing animals to explore the sand. Trials were filmed with GoPro HERO 7 cameras (GoPro, Inc.), outside the behavioral tank. Trials were run for 30 min, then the tanks were reset by removing excess mussels and agitating the sand. Ten trials in succession were performed for each tank, ensuring that the mussel pieces changed position in the tank each time.

GoPro videos were uploaded and played back in Windows Media Player to assess capture success. Capture success was defined as a fish locating the mussel, evidenced by mussel consumption or a ‘head snap’ in which fish open their mouths and snap in the direction of the prey item. Each group tank had two mussel pieces or ‘chances of success’ per trial. Data were compiled and visualized in Graphpad Prism. A Wilcoxon rank-sum test with Benjamini-Hochberg correction for false discovery rate was used to assay significance.

#### Protein alignments

Tbx3a protein sequences from *Gasterosteus aculeatus* (stickleback; GenBank GCA_016920845.1_GAculeatus_UGA_version5 genome assembly), *Oryzias latipes* (medaka; GenBank GCA_002234675.1ASM223467v1 genome assembly) and *Danio rerio* (zebrafish; GenBank GCA_000002035.4_GRCz11 genome assembly) were downloaded from Ensembl (https://useast.ensembl.org/index.html). Protein sequences from the *Prionotus carolinus* and *Prionotus evolans* annotations were used. DNA sequences of exons were confirmed by visual inspection of RNA-seq read alignment to the respective genomes in the Integrated Genomics Viewer (IGV, version 2.16.2) (*N* = 4 samples from *P. carolinus* and *N* = 3 samples from *P. evolans*). Protein sequences were aligned in Geneious (version 2023.1.2) using MAFFT alignment (version 7.490,^59^) and visually inspected for amino acids that were completely conserved in stickleback, medaka, and zebrafish, and altered in both sea robin species.

#### Skeletal preparations

Skeletal preparations were performed according to standard protocols.^60^ In brief, fish were euthanized and fixed in 10% Neutral buffered formalin (NBF). Samples were rinsed 4x in distilled water overnight while rocking. The next day, fish were stained in Alcian blue, rehydrated in a series of ethanol washes, and rinsed in 30% saturated sodium borate. Samples were incubated in 0.12% porcine trypsin in 30% saturated sodium borate for 3–6 h at room temperature. Trypsin solution was removed and samples were washed twice in 2% potassium hydroxide (KOH) before being moved into a 0.002% Alizarin red solution in 2% KOH overnight. The next day, samples were bleached with 30% hydrogen peroxide in a 3 part 0.5% KOH and 1 part glycerol solution for up to 8 h until removal of pigment was sufficient. Samples were then transferred to 100% glycerol with thymol crystals through a 0.5% KOH: glycerol series (1:1, 1:3, 100% glycerol). Leg bone segments were counted under a dissecting microscope. Legs and fins were dissected from the body and images were taken on a Zeiss Stemi SV 11. FIJI was used to measure VHP width and total width, and the standard length of all samples was measured using a ruler. A linear regression model in R version 4.2.2 was used to calculate the residuals of VHP width and total width. *N* = 3 control animals (*n* = 3 legs) and *N* =3 *tbx3a* crispants (*n* = 5 legs) were used in analysis. A Shapiro-Wilk test for normality and a Welch’s t test were used to calculate significance.

### QUANTIFICATION AND STATISTICAL ANALYSIS

Data were analyzed using R (versions specified in accompanying code) and Graphpad Prism (v10). N represented independent individuals while n represented total samples. Statistical tests used in analyses included a Wilcoxon rank-sum test, a Wilcoxon rank-sum test with Benjamini-Hochberg correction for false discovery rate, a Fisher’s exact test, a Fisher’s exact test with FDR correction, a Shapiro-Wilk test for normality, a two-sample t test, a Welch’s t test, a Welch’s t test with Benjamini-Hochberg FDR correction, and an ANOVA with Tukey’s HSD post hoc test as described in the STAR Methods section. Boxplot elements are defined as: center line, median; box limits, interquartile range (IQR); whiskers, 1.5 × IQR.

## Supplementary Material

Supplemental Ancient developmental genes underlie evolutionary novelties in walking fish

## Figures and Tables

**KEY RESOURCES TABLE:** 

REAGENT or RESOURCE	SOURCE	IDENTIFIER

Chemicals, peptides, and recombinant proteins		
Cas9–2NLS protein	QB3 MacroLab, University of California - Berkeley	N/A
Critical commercial assays		
RNAscope^™^ Multiplex Fluorescent detection kit v2	Advanced Cell Diagnostics	323110
DNeasy blood and tissue kit	Qiagen	69506
Allprep DNA/RNA micro kit	Qiagen	80284
2x Phusion Green Hot Start II High-Fidelity PCR Master Mix	ThermoFisher Scientific	F566
QIAquick PCR purification kit	Qiagen	28106
Qubit DNA High Sensitivity kit	Invitrogen	Q32854
RNeasy micro kit	Qiagen	74034
Qubit RNA High Sensitivity Kit	Invitrogen	Q32855
SMART-Seq^®^ v4 Ultra^®^ Low Input RNA Kit for Sequencing	Takara	634890; 634888
Nextera XT DNA library preparation kit	Illumina	FC-131–1024; FC-131–1096
Deposited data		
Raw sequencing data	This paper; SRA	SRA: BioProject PRJNA1136006
Data and code	This paper; Figshare	Figshare: https://doi.org/10.6084/m9.figshare.26053234
Experimental models: Organisms/strains		
*Prionotus carolinus*	Adults obtained from the Marine Biological Laboratory; juveniles obtained through *in vitro* fertilization	N/A
*Prionotus evolans*	Adults obtained from the Marine Biological Laboratory; juveniles obtained through *in vitro* fertilization	N/A
*Prionotus carolinus / Prionotus evolans* hybrids	Obtained through *in vitro* fertilization of a *P. carolinus* and *P. evolans* adult	N/A
Oligonucleotides		
*tbx3a* sgRNA; DK_ALH_9: AATTAATACG ACTCACTATAGGGCACCTCACTTT GAACGGGTTTTAGAGCTAGAAATAGC	IDT	N/A
*tbx3a* sgRNA; DK_ALH_10: AATTAATA CG ACTCACTATAGGACTTGTATCTGC AGTCGTGTTTTAGAGCTAGAAATAGC	IDT	N/A
*slc24a5* guide 3: AATTAATACGACTCA CTATAGGCAGGCAGTCCCTGAACAG GTTTTAGAGCTAGAAATAGC	IDT	N/A
*slc24a5* genotyping F primer; DK_ALH_223: ACCTGCTAGGAATCTGTGCTG	IDT	N/A
*slc24a5* genotyping R primer; DK_ALH_224: TGCGCTGTACTGTCATTGACT	IDT	N/A
*tbx3a* genotyping F primer; DK_ALH_18: AACATAAAGCCGATGCAGCG	IDT	N/A
*tbx3a* genotyping R primer; DK_ALH_17 TCCATGCTTGTCGGAGATGT	IDT	N/A
Software and algorithms		
Liftoff	Shumate and Salzberg^45^	https://github.com/agshumate/Liftoff
FastQC Version 0.11.9	N/A	https://www.bioinformatics.babraham.ac.uk/projects/fastqc/
Cutadapt Version 1.18	Martin^46^	https://cutadapt.readthedocs.io/en/stable/
STAR Version 2.7.9a; Version 2.7.10b	Dobin et al.^47^	https://github.com/alexdobin/STAR
DESeq2	Love et al.^48^	https://bioconductor.org/packages/release/bioc/html/DESeq2.html
R	R Development Core Team	https://www.r-project.org/
Gene Ontology Resource Site	N/A	https://geneontology.org/
FIJI	Schindelin et al.^49^	https://fiji.sc/
Graphpad Prism Version 10	N/A	https://www.graphpad.com/
IGV Version 2.16.2	N/A	https://igv.org/doc/desktop/
Geneious Version 2023.1.2	N/A	https://www.geneious.com/
MAFFT Version 7.490	N/A	https://mafft.cbrc.jp/alignment/software/
Salmon Version 1.10.3	Patro et al.^50^	https://github.com/COMBINE-lab/salmon

## Highlights

- Sea robins are fish that walk and probe substrate with leg-like structures
- Transcriptomics identifies ancient genes controlling leg formation from fins
- Genome editing of *tbx3a* disrupts legs, nervous system structures, and behavior
- Analysis of sea robin hybrids reveals genes underlying trait differences

## References

[1] Stern DL, and Orgogozo V (2008). The loci of evolution: how predictable is genetic evolution? Evolution 62, 2155–2177. 10.1111/j.1558-5646.2008.00450.x. 18616572 PMC2613234

[2] Sucena E, and Stern DL (2000). Divergence of larval morphology between Drosophila sechellia and its sibling species caused by cis-regulatory evolution of ovo/shaven-baby. Proc. Natl. Acad. Sci. USA 97, 4530–4534. 10.1073/pnas.97.9.4530. 10781057 PMC18269

[3] Gross JB, Borowsky R, and Tabin CJ (2009). A novel role for Mc1r in the parallel evolution of depigmentation in independent populations of the cavefish Astyanax mexicanus. PLoS Genet. 5, e1000326. 10.1371/journal.pgen.1000326. 19119422 PMC2603666

[4] Leal F, and Cohn MJ (2016). Loss and re-emergence of legs in snakes by modular evolution of sonic hedgehog and HOXD enhancers. Curr. Biol. 26, 2966–2973. 10.1016/j.cub.2016.09.020. 27773569

[5] Colosimo PF, Hosemann KE, Balabhadra S, Villarreal G Jr., Dickson M, Grimwood J, Schmutz J, Myers RM, Schluter D, and Kingsley DM (2005). Widespread parallel evolution in sticklebacks by repeated fixation of ectodysplasin alleles. Science 307, 1928–1933. 10.1126/science.1107239. 15790847

[6] Chan YF, Marks ME, Jones FC Jr., Villarreal G Jr., Shapiro MD, Brady SD, Southwick AM, Absher DM, Grimwood J, Schmutz J, (2010). Adaptive evolution of pelvic reduction in sticklebacks by recurrent deletion of a pitx1 enhancer. Science 327, 302–305. 10.1126/science.1182213. 20007865 PMC3109066

[7] Gehrke AR, and Shubin NH (2016). Cis-regulatory programs in the development and evolution of vertebrate paired appendages. Semin. Cell Dev. Biol. 57, 31–39. 10.1016/J.SEMCDB.2016.01.015. 26783722 PMC5360378

[8] Adachi N, Robinson M, Goolsbee A, and Shubin NH (2016). Regulatory evolution of Tbx5 and the origin of paired appendages. Proc. Natl. Acad. Sci. USA 113, 10115–10120. 10.1073/pnas.1609997113. 27503876 PMC5018757

[9] Nakamura T, Gehrke AR, Lemberg J, Szymaszek J, and Shubin NH (2016). Digits and fin rays share common developmental histories. Nature 537, 225–228. 10.1038/nature19322. 27533041 PMC5161576

[10] Letelier J, De La Calle-Mustienes E, Pieretti J, Naranjo S, Maeso I, Nakamura T, Pascual-Anaya J, Shubin NH, Schneider I, Martinez-Morales JR, and Gómez-Skarmeta JL. (2018). A conserved Shh cis-regulatory module highlights a common developmental origin of unpaired and paired fins. Nat. Genet. 50, 504–509. 10.1038/s41588-018-0080-5. 29556077 PMC5896732

[11] Sackton TB, Grayson P, Cloutier A, Hu Z, Liu JS, Wheeler NE, Gardner PP, Clarke JA, Baker AJ, Clamp M, and Edwards SV (2019). Convergent regulatory evolution and loss of flight in paleognathous birds. Science 364, 74–78. 10.1126/science.aat7244. 30948549

[12] Wang Y, Zhang C, Wang N, Li Z, Heller R, Liu R, Zhao Y, Han J, Pan X, Zheng Z, (2019). Genetic basis of ruminant headgear and rapid antler regeneration. Science 364, eaav6335. 10.1126/SCIENCE.AAV6335. 31221830

[13] Marcovitz A, Turakhia Y, Chen HI, Gloudemans M, Braun BA, Wang H, and Bejerano G (2019). A functional enrichment test for molecular convergent evolution finds a clear protein-coding signal in echolocating bats and whales. Proc. Natl. Acad. Sci. USA 116, 21094–21103. 10.1073/PNAS.1818532116. 31570615 PMC6800341

[14] Finger TE (2000). Ascending spinal systems in the fish, Prionotus carolinus. J. Comp. Neurol. 422, 106–122. 10.1002/(SICI)1096-9861(20000619)422:1<106::AID-CNE7>3.0.CO;2-T. 10842221

[15] Hale ME, Galdston S, Arnold BW, and Song C (2022). The water to land transition submerged: multifunctional design of pectoral fins for use in swimming and in association with underwater substrate. Integr. Comp. Biol. 62, 908–921. 10.1093/ICB/ICAC061. 35652788 PMC9617210

[16] Finger TE (1982). Somatotopy in the representation of the pectoral fin and legs in the spinal cord of the sea robin, Prionotus carolinus. Bio Bull 163, 154–161. 10.2307/1541505.

[17] Harris J (2013). The comparative morphology of the pectoral free rays in scorpaenoid fishes (perciformes: Scorpaenoidea). Master’s Theses. https://ecommons.luc.edu/luc_theses/1457/. 10.1002/jmor.21597 37313767

[18] Allard CAH, Herbert AL, Krueger SP, Liang Q, Walsh BL, Rhyne AL, Gourlay AN, Seminara A, Baldwin MW, Kingsley DM, and Bellono NW (2024). Evolution of novel sensory organs in fish with legs. Curr. Bio. 34, 10.1016/j.cub.2024.08.014. PMC11552235 39332400

[19] Smith WL, Everman E, and Richardson C (2018). Phylogeny and taxonomy of Flatheads, Scorpionfishes, Sea Robins, and Stonefishes (Percomorpha: Scorpaeniformes) and the evolution of the lachrymal saber. Copeia 106, 94–119. 10.1643/CG-17-669.

[20] Taft N, Harris J, and Grande TC (2023). The comparative morphology of the musculature controlling the pectoral free rays in scorpaenoid fishes. J. Morphol. 284, e21597. 10.1002/jmor.21597. 37313767

[21] Yuschak P, and Lund WA (1984). Eggs, larvae and osteological development of the Northern searobin, Prionotus carolinus (Pisces, Triglidae). J. Northw. Atl. Fish. Sci. 5, 1–15. 10.2960/J.v5.a1.

[22] Yuschak P (1985). Fecundity, eggs, larvae and osteological development of the Striped searobin, (Prionotus evolans) (Pisces, Triglidae). J. Northw. Atl. Fish. Sci. 6, 65–85. 10.2960/j.v6.a7.

[23] Petersen JC, and Ramsay JB (2020). Walking on chains: the morphology and mechanics behind the fin ray derived limbs of sea-robins. J. Exp. Biol. 223, jeb227140. 10.1242/jeb.227140. 32709626

[24] Ahn D, and Ho RK (2008). Tri-phasic expression of posterior Hox genes during development of pectoral fins in zebrafish: implications for the evolution of vertebrate paired appendages. Dev. Biol. 322, 220–233. 10.1016/j.ydbio.2008.06.032. 18638469

[25] Tarchini B, and Duboule D (2006). Control of hoxd genes’ collinearity during early limb development. Dev. Cell 10, 93–103. 10.1016/j.devcel.2005.11.014. 16399081

[26] Khan SF, Damerell V, Omar R, Du Toit M, Khan M, Maranyane HM, Mlaza M, Bleloch J, Bellis C, Sahm BDB, (2020). The roles and regulation of TBX3 in development and disease. Gene 726, 144223. 10.1016/j.gene.2019.144223. 31669645 PMC7108957

[27] Yonei-Tamura S, Tamura K, Tsukui T, and Izpisú a Belmonte, J.C. (1999). Spatially and temporally-restricted expression of two T-box genes during zebrafish embryogenesis. Mech. Dev. 80, 219–221. 10.1016/S0925-4773(98)00219-6. 10072792

[28] Kuijper S, Beverdam A, Kroon C, Brouwer A, Candille S, Barsh G, and Meijlink F (2005). Genetics of shoulder girdle formation: roles of Tbx15 and aristaless-like genes. Development 132, 1601–1610. 10.1242/dev.01735. 15728667

[29] Begemann G, Gibert Y, Meyer A, and Ingham PW (2002). Cloning of zebrafish T-box genes tbx15 and tbx18 and their expression during embryonic development. Mech. Dev. 114, 137–141. 10.1016/S0925-4773(02)00040-0. 12175500

[30] Zhang J, Wagh P, Guay D, Sanchez-Pulido L, Padhi BK, Korzh V, Andrade-Navarro MA, and Akimenko M-A (2010). Loss of fish actinotrichia proteins and the fin-to-limb transition. Nature 466, 234–237. 10.1038/nature09137. 20574421

[31] Rallis C, Bruneau BG, Del Buono J, Seidman CE, Seidman JG, Nissim S, Tabin CJ, and Logan MPO (2003). Tbx5 is required for forelimb bud formation and continued outgrowth. Development 130, 2741–2751. 10.1242/dev.00473. 12736217

[32] Bamshad M, Lin RC, Law DJ, Watkins WC, Krakowiak PA, Moore ME, Franceschini P, Lala R, Holmes LB, Gebuhr TC, (1997). Mutations in human TBX3 alter limb, apocrine and genital development in Ulnar-mammary syndrome. Nat. Genet. 16, 311–315. 10.1038/NG0797-311. 9207801

[33] Bamshad M, Le T, Watkins WS, Dixon ME, Kramer BE, Roeder AD, Carey JC, Root S, Schinzel A, Van Maldergem L, (1999). The spectrum of mutations in TBX3: genotype/phenotype relationship in Ulnar-mammary syndrome. Am. J. Hum. Genet. 64, 1550–1562. 10.1086/302417. 10330342 PMC1377898

[34] Wucherpfennig JI, Miller CT, and Kingsley DM (2019). Efficient CRISPR-Cas9 editing of major evolutionary loci in sticklebacks. Evol. Ecol. Res. 20, 107–132. https://www.ncbi.nlm.nih.gov/pmc/articles/PMC8664273/. 34899072 PMC8664273

[35] Masselink W (2021). Crispants take the spotlight. Lab Anim. 50, 95–96. 10.1038/s41684-021-00739-6. 33737756

[36] Sur A, Wang Y, Capar P, Margolin G, and Farrell JA (2023). Single-cell analysis of shared signatures and transcriptional diversity during zebrafish development. Preprint at bioRxiv. 10.1101/2023.03.20.533545. PMC11181902 37995681

[37] Imsland F, McGowan K, Rubin CJ, Henegar C, Sundströ m E., Berglund J., Schwochow D., Gustafson U., Imsland P., Lindblad-Toh K., (2016). Regulatory mutations in TBX3 disrupt asymmetric hair pigmentation that underlies Dun camouflage color in horses. Nat. Genet. 48, 152–158. 10.1038/ng.3475. 26691985 PMC4731265

[38] Hill MS, Vande Zande P, and Wittkopp PJ (2021). Molecular and evolutionary processes generating variation in gene expression. Nat. Rev. Genet. 22, 203–215. 10.1038/s41576-020-00304-w. 33268840 PMC7981258

[39] Daane JM, Blum N, Lanni J, Boldt H, Iovine MK, Higdon CW, Johnson SL, Lovejoy NR, and Harris MP (2021). Modulation of bioelectric cues in the evolution of flying fishes. Curr. Biol. 31, 5052–5061.e8. 10.1016/j.cub.2021.08.054. 34534441 PMC9172250

[40] Newton AH, Williams SM, Phipson B, Pask AJ, Major AT, and Smith CA (2023). Heterotopic reduction of forelimb progenitors underpins development of the vestigial emu wing; implications for vertebrate limb evolution. Preprint at bioRxiv. 10.1101/2022.11.23.516993.

[41] Yuan Y, Sun D-M, Qin T, Mao S-Y, Zhu W-Y, Yin Y-Y, Huang J, Heller R, Li Z-P, Liu J-H, and Qiu Q (2022). Single-cell transcriptomic landscape of the sheep rumen provides insights into physiological programming development and adaptation of digestive strategies. Zool. Res. 43, 634–647. 10.24272/J.ISSN.2095-8137.2022.086. 35838034 PMC9336438

[42] Pendyala M, Stephen SJ, Vashishth D, Blaber EA, and Chan DD (2023). Loss of hyaluronan synthases impacts bone morphology, quality, and mechanical properties. Bone 172, 116779. 10.1016/j.bone.2023.116779. 37100359

[43] Tambalo M, Mitter R, and Wilkinson DG (2020). A single cell transcriptome atlas of the developing zebrafish hindbrain. Development 147, dev184143. 10.1242/dev.184143. 32094115 PMC7097387

[44] Kölsch Y., Hahn J., Sappington A., Stemmer M., Fernandes AM., Helmbrecht TO., Lele S., Butrus S., Laurell E., Arnold-Ammer I., (2021). Molecular classification of zebrafish retinal ganglion cells links genes to cell types to behavior. Neuron 109, 645–662.e9. 10.1016/j.neuron.2020.12.003. 33357413 PMC7897282

[45] Shumate A, and Salzberg SL (2021). Liftoff: accurate mapping of gene annotations. Bioinformatics 37, 1639–1643. 10.1093/bioinformatics/btaa1016. 33320174 PMC8289374

[46] Martin M (2011). Cutadapt removes adapter sequences from high-throughput sequencing reads. EMBnet. j. 17, 10–12. 10.14806/ej.17.1.200.

[47] Dobin A, Davis CA, Schlesinger F, Drenkow J, Zaleski C, Jha S, Batut P, Chaisson M, and Gingeras TR (2013). STAR: ultrafast universal RNA-seq aligner. Bioinformatics 29, 15–21. 10.1093/BIOINFORMATICS/BTS635. 23104886 PMC3530905

[48] Love MI, Huber W, and Anders S (2014). Moderated estimation of fold change and dispersion for RNA-seq data with DESeq2. Genome Biol. 15, 550. 10.1186/s13059-014-0550-8. 25516281 PMC4302049

[49] Schindelin J, Arganda-Carreras I, Frise E, Kaynig V, Longair M, Pietzsch T, Preibisch S, Rueden C, Saalfeld S, Schmid B, (2012). Fiji: an open-source platform for biological-image analysis. Nat. Methods 9, 676–682. 10.1038/nmeth.2019. 22743772 PMC3855844

[50] Patro R, Duggal G, Love MI, Irizarry RA, and Kingsford C (2017). Salmon: fast and bias-aware quantification of transcript expression using dual-phase inference. Nat. Methods 14, 417–419. 10.1038/nmeth.4197. 28263959 PMC5600148

[51] Guide for the Care and Use of Laboratory Animals, Eighth Edition (2010) National Academies Press.

[52] Tlusty MF, Baylina N, Rhyne AL, Brown C, and Smith M (2017). Public Aquaria. In Marine Ornamental Species Aquaculture (John Wiley & Sons, Ltd), pp. 611–622. 10.1002/9781119169147.ch27a.

[53] Putnam NH, O’Connell BL, Stites JC, Rice BJ, Blanchette M, Calef R, Troll CJ, Fields A, Hartley PD, Sugnet CW, (2016). Chromosome-scale shotgun assembly using an in vitro method for long-range linkage. Genome Res. 26, 342–350. 10.1101/gr.193474.115. 26848124 PMC4772016

[54] Simã o FA., Waterhouse RM., Ioannidis P., Kriventseva EV., and Zdobnov EM. (2015). BUSCO: assessing genome assembly and annotation completeness with single-copy orthologs. Bioinformatics 31, 3210–3212. 10.1093/BIOINFORMATICS/BTV351. 26059717

[55] Babraham Bioinformatics FastQC A Quality Control tool for High Throughput Sequence Data https://www.bioinformatics.babraham.ac.uk/projects/fastqc/.

[56] Thorndike RL (1953). Who belongs in the family? Psychometrika 18, 267–276. 10.1007/BF02289263.

[57] Rousseeuw PJ (1987). Silhouettes: A graphical aid to the interpretation and validation of cluster analysis. J. Comput. Appl. Math. 20, 53–65. 10.1016/0377-0427(87)90125-7.

[58] Song JHT, Grant RL, Behrens VC, Kučka M, Roberts Kingman GA, Soltys V, Chan YF, and Kingsley DM (2021). Genetic studies of human-chimpanzee divergence using stem cell fusions. Proc. Natl. Acad. Sci. USA 118, e2117557118. 10.1073/PNAS.2117557118. 34921118 PMC8713981

[59] Katoh K, and Standley DM (2013). MAFFT multiple sequence alignment software version 7: improvements in performance and usability. Mol. Biol. Evol. 30, 772–780. 10.1093/molbev/mst010. 23329690 PMC3603318

[60] Rigueur D, and Lyons KM (2014). Whole-mount skeletal staining. Methods Mol. Biol. 1130, 113–121. 10.1007/978-1-62703-989-5_9. 24482169 PMC5384832

