# Supplementary material for: Ancient developmental genes underlie evolutionary novelties in walking fish
Source: Curr Biol. Author manuscript; Available in PMC 2024 Nov 11. (PMC11552234; doi:10.1016/j.cub.2024.08.042)
Supplement: Supplemental Ancient developmental genes underlie evolutionary novelties in walking fish [file NIHMS2029048-supplement-Supplemental_Ancient_developmental_genes_underlie_evolutionary_novelties_in_walking_fish.pdf]

Current Biology, Volume *34*

Supplemental Information

Ancient developmental genes underlie evolutionary novelties in walking fish

Amy L. Herbert, Corey A.H. Allard, Matthew J. McCoy, Julia I. Wucherpfennig, Stephanie P. Krueger, Heidi I. Chen, Allex N. Gourlay, Kohle D. Jackson, Lisa A. Abbo, Scott H. Bennett, Joshua D. Sears, Andrew L. Rhyne, Nicholas W. Bellono, and David M. Kingsley

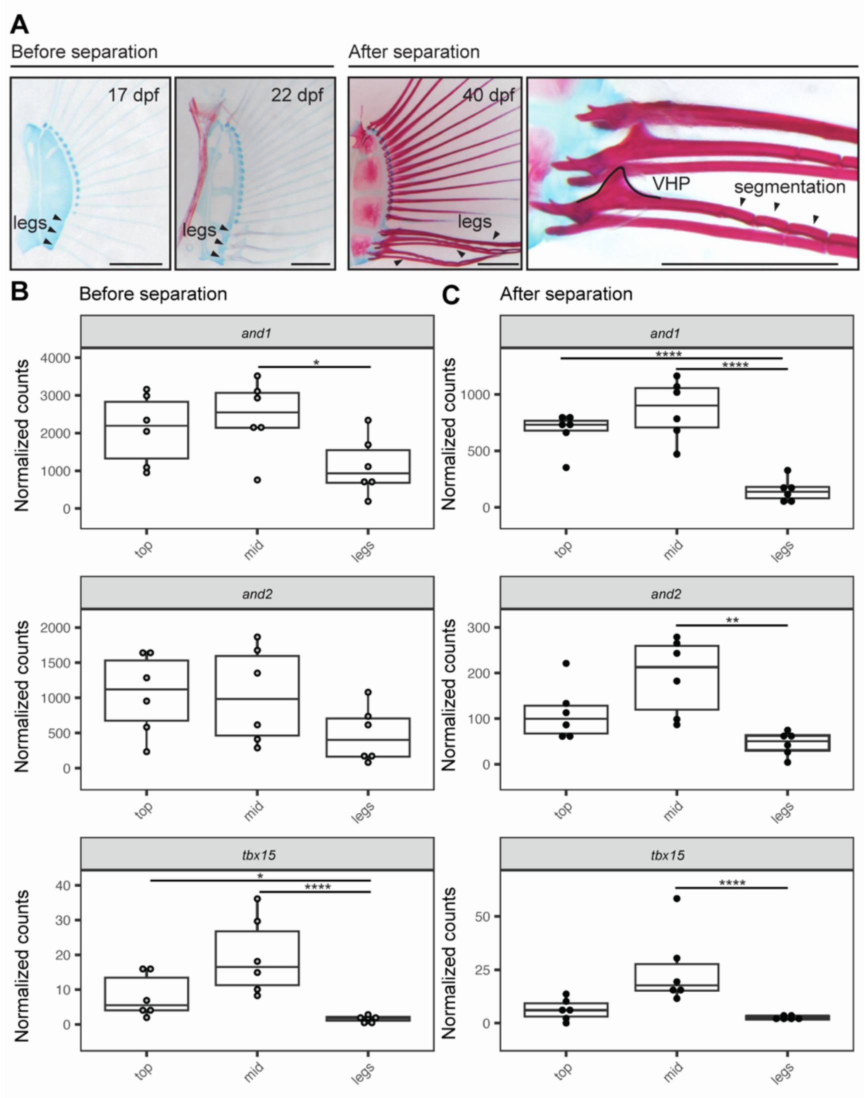

Figure S1. Morphological changes and gene expression differences during leg and fin ray development, related to Figure 1. (**A**) Before separation, pectoral rays exhibit distal radials (one per ray) which are not present at the base of the developing leg rays. By 40 dpf, separated legs show larger widths and the distinctive ventral hemitrichial process (VHP) that forms at the base of one hemitrich in each leg. (**B**) Boxplot showing normalized gene counts from RNA-seq analysis of actinodin fibril genes (*and1*, *and2*), and *tbx15* in developing legs and fins, including the top three fin rays (top), middle fin rays (mid) and the legs (leg) before leg separation. (**C**) Normalized counts of genes after leg separation. Exact *P-adjusted* values: *padj* = 0.02 (**B**, *and1*), *padj* = 0.02 (**B**, *tbx15* top vs. legs), *padj* = 3.30e-6 (**B,** *tbx15* mid vs. legs), *padj* = 7.68e-12 (**C**, *and1* top vs. legs), *padj* = 9.73e-15 (**C**, *and1* mid vs. legs), *padj* = 0.0018 (**C**, *and2*), *padj* = 1.92e-06 (**C**, *tbx15*). N = 6 animals before separation and N = 6 animals after separation were used for RNA-seq analysis. Scalebars, 0.5 mm (before separation), 1 mm (after separation).

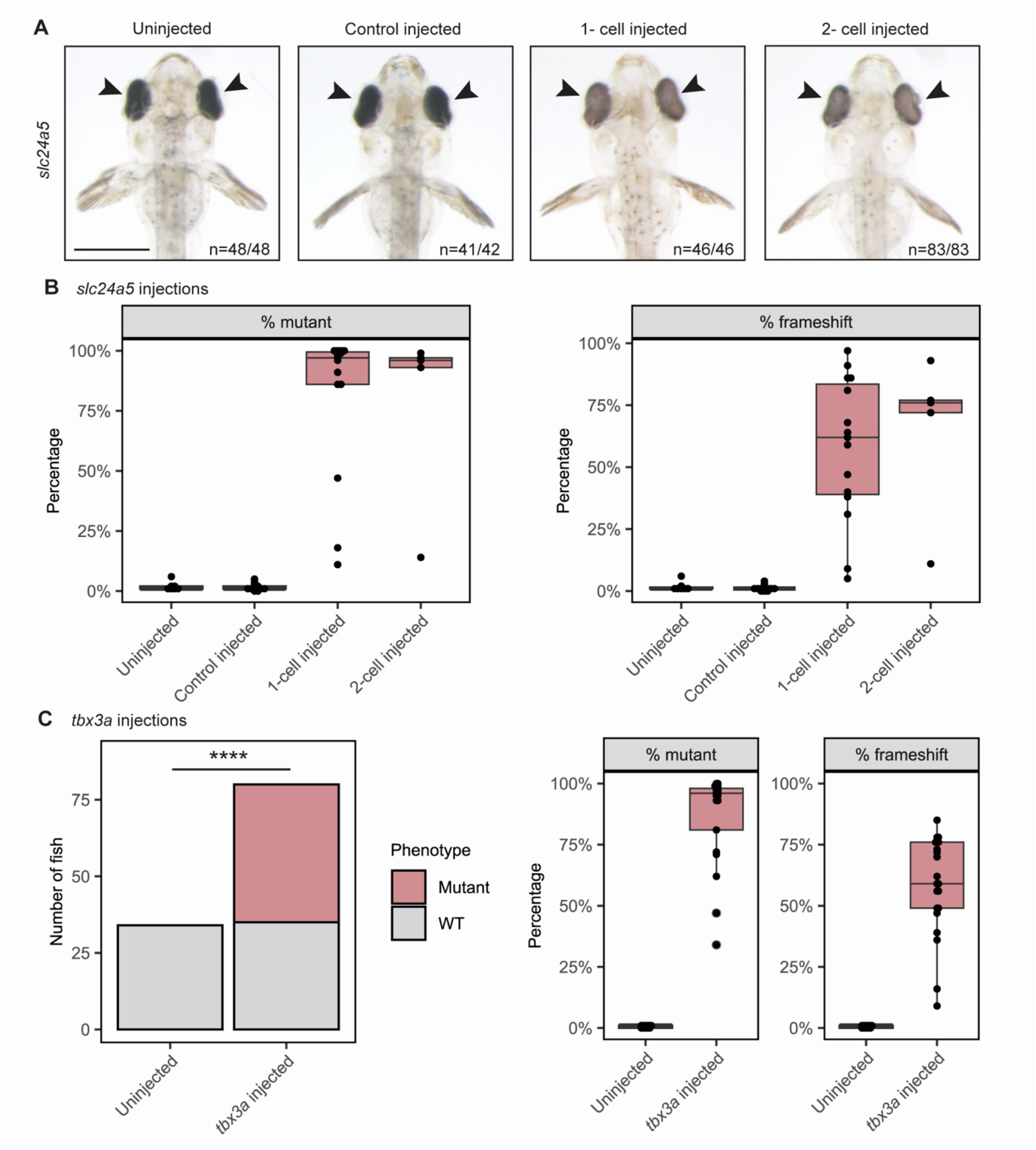

Figure S2. CRISPR-Cas9 genome editing of sea robin genes, related to Figure 2. (**A**) Targeting of the pigment gene *slc24a5* at the 1-2 cell stage reduced pigmentation in larval eyes (arrowheads) of injected animals compared to uninjected or control injected siblings. (**B**) *Slc24a5* crispant larvae showed a high percentage of both mutant and frameshift reads compared to uninjected and control injected larvae. (**C**) Quantification of crispant phenotypes in *tbx3a* injected animals. There was again a high percentage of mutant and frameshift reads from sequenced crispants compared to uninjected animals. Fisher's exact test used for calculating significance. Exact *p-value:* $p = 5.29e\text{-}10$ (**C**). Scale bar, 1 mm (**A**).

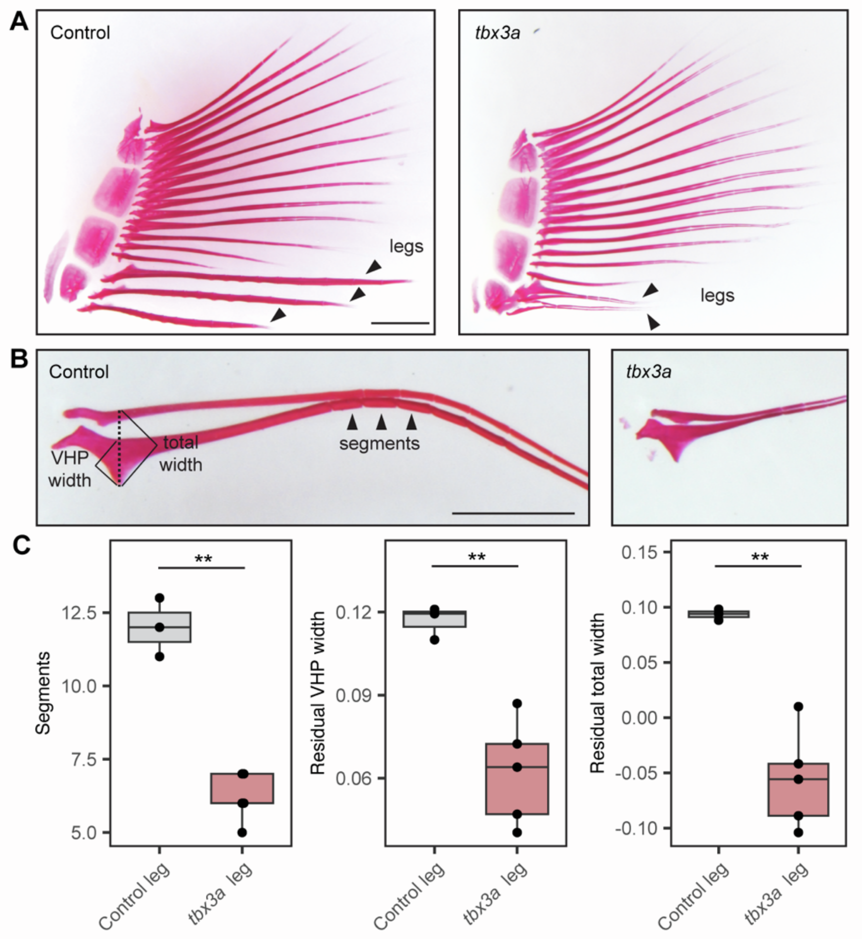

Figure S3. Morphology of *tbx3a* legs, related to Figure 2. (**A**) A skeletal preparation from a control animal shows three robust legs, in contrast to the two reduced legs found in a *tbx3a* crispant. (**B-C**) The number of segments and measurements of VHP width and total width were significantly decreased in *tbx3a* crispants compared to control animals. VHP width and total width were regressed against the standard length of the animals. A Welch's t test was used for calculating significance. Exact *p-values:* $p = 0.0015$ (segments), $p = 0.0017$ (residual VHP width), $p = 0.0015$ (residual total width). N = 3 control animals (n = 3 legs) and N = 3 *tbx3a* crispant animals (n = 5 reduced legs) were used for analysis. Scalebars, 1 mm (**A, B**).

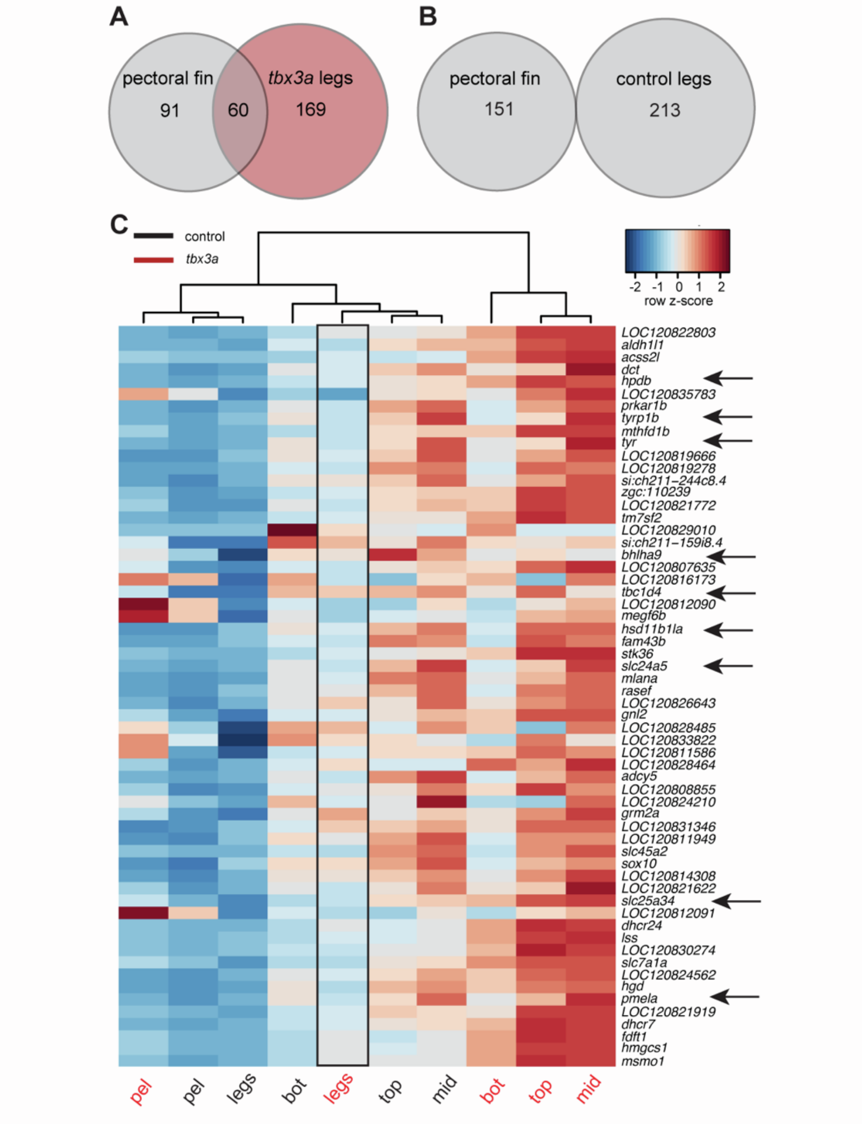

Figure S4. Gene expression in *tbx3a* crispants before separation, related to Figure 2. (**A**) Venn diagram of overlapping genes upregulated in pectoral fins compared to control legs and *tbx3a* crispant legs compared to control legs (*padj* < 0.1). (**B**) Venn diagram showing no overlap between genes upregulated in pectoral fins and genes upregulated in control legs compared to *tbx3a* crispant legs (*padj* < 0.1). (**C**) Heatmap of the 60 intersecting genes identified in (**A**). Arrows point to fin and pigment genes that cluster *tbx3a* crispant legs with control legs.

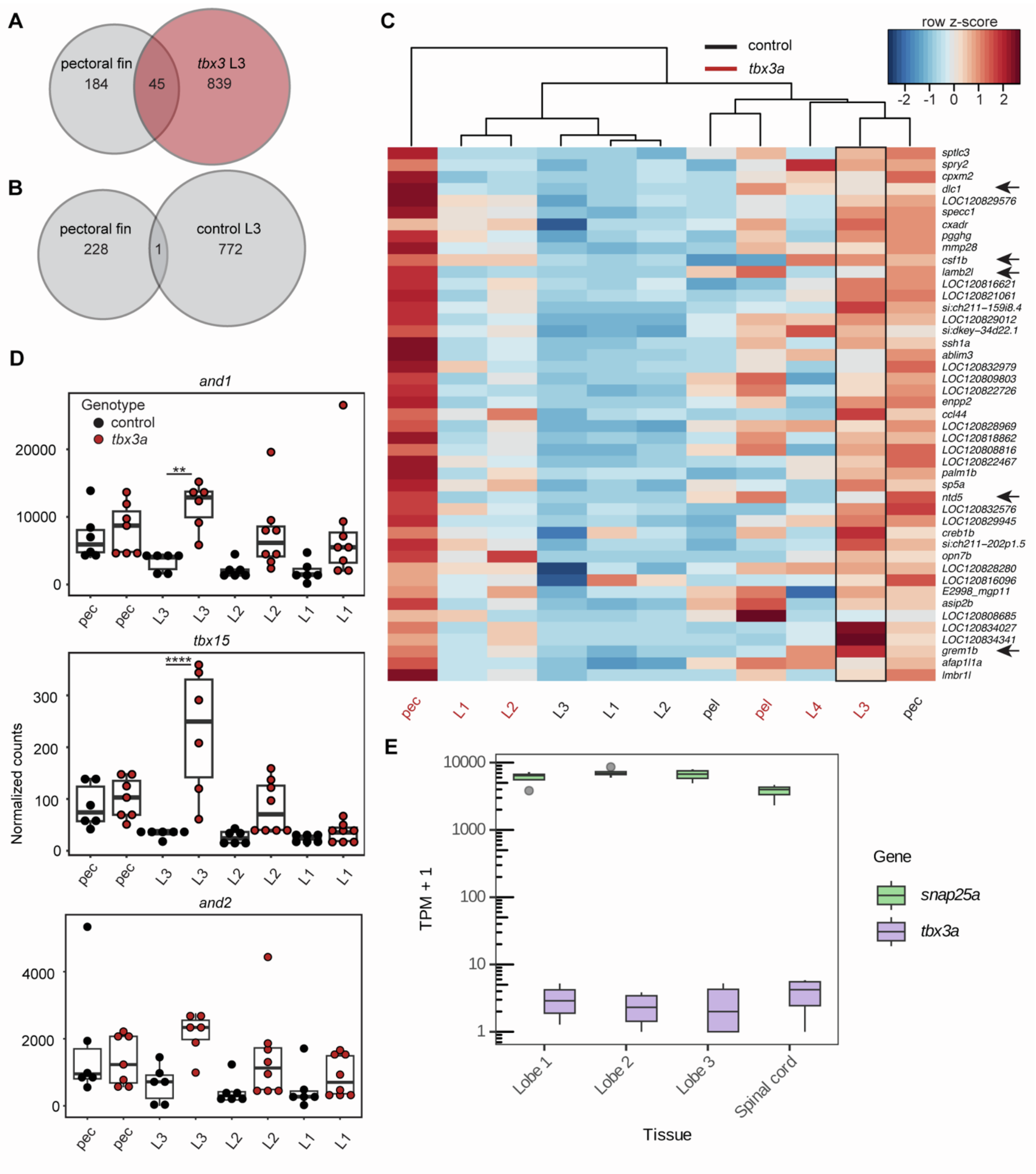

Figure S5. Gene expression in *tbx3a* crispants and WT lobes after separation, related to Figure 2. (**A**) Venn diagram of 45 overlapping genes upregulated in the pectoral fin compared to control leg 3 and *tbx3a* crispant leg 3 compared to control legs (cutoff set at *padj* < 0.1). (**B**) Venn diagram showing one gene is upregulated in pectoral fins and in control legs compared to *tbx3a* crispant leg 3 (cut off set at *padj* < 0.1). (**C**) Heatmap of the 45 intersecting genes identified in (**A**), with arrows pointing to fin genes. (**D**) Boxplots showing normalized gene counts of *and1* and *tbx15*. *And1* and *tbx15* expression is upregulated in *tbx3a* crispant leg 3 compared to control leg 3. *And2* counts show similar trends but do not rise to significance. Exact

p-adjusted values: *padj* = 0.007 (**D**, *and1*, *tbx3a* crispant leg 3 vs. control leg 3), *padj* = 2.27e-9 (**D**, *tbx15*, *tbx3a* crispant leg 3 vs. control leg 3). N = 6 control animals and N = 8 *tbx3a* crispants (N = 7 *tbx3a* pec fins). N = 2/8 crispant animals had two legs. (**E**) The neuronal marker *snap25a* is highly expressed in adult sea robin lobes and in the underlying spinal cord. In contrast, there is very low but detectable expression of *tbx3a* in both lobes and spinal cord. N = 4 animals. TPM = transcripts per million.

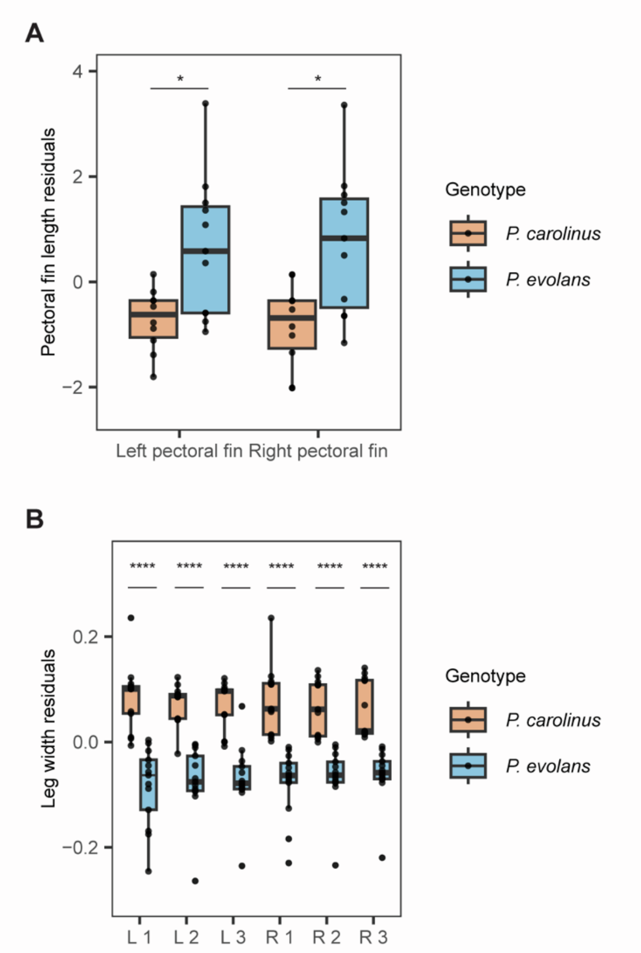

Figure S6. Species-specific leg and fin differences, related to Figure 3. (**A**) Left and right pectoral fin length residuals of *P. evolans* are significantly increased compared to *P. carolinus*. (**B**) *P. carolinus* legs are significantly wider than *P. evolans* for every leg. Leg width regressed against the standard length (SL) of the fish. Box and whisker plots show the median at the center line and whiskers in the interquartile range (**A, B**). Significance determined by Wilcoxon rank-sum test with Benjamini-Hochberg correction for false discovery rate. In all graphs, *$p < 0.05$, ****$p < 0.0001$. N = 10 *P. carolinus* and N = 11 *P. evolans* (**A**, pectoral fin measurements). N = 13 *P. carolinus* and N = 13 *P. evolans* (**B**, leg width measurements).

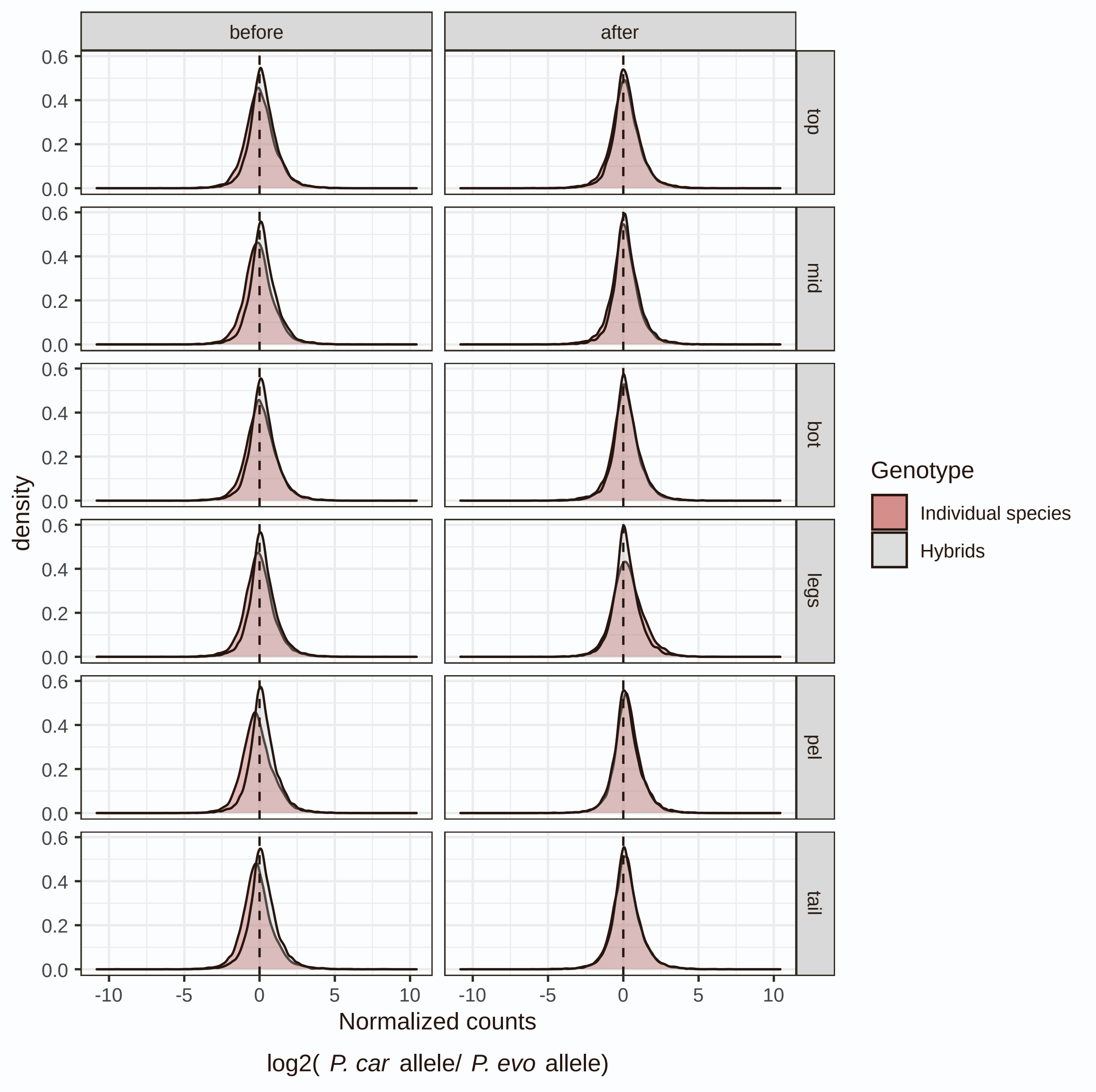

Figure S7. Species-specific differential gene expression, related to Figure 3. Density plots of normalized counts for *P. carolinus* versus *P. evolans* alleles in individual species (red) and F1 hybrids (gray) across different tissues (top, mid, bot, legs, pel, and tail) and developmental stages (before and after leg separation). The distribution of allele-specific expression in F1 hybrids is slightly biased toward *P. carolinus* alleles, which may reflect paternal allele bias or species-specific expression bias as has been noted in other studies [S1,S2].

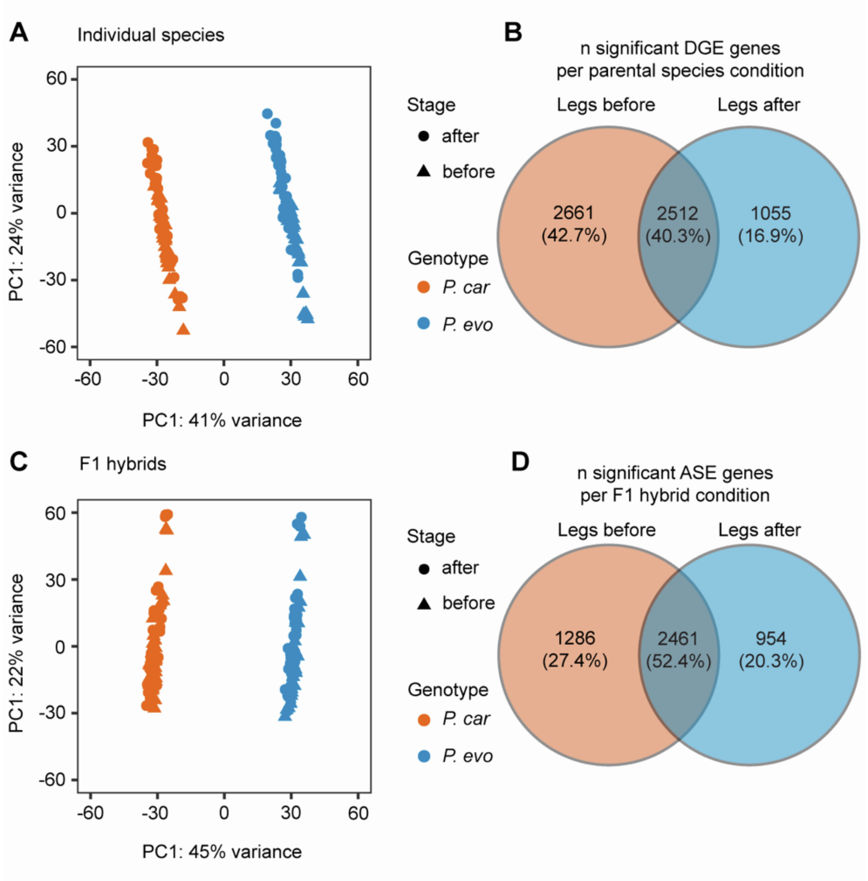

Figure S8. Gene expression in individual species and hybrids, related to Figure 3. (**A**) Principal component analysis of legs before and after separation in *P. carolinus* (*P. car*) and *P. evolans* (*P. evo*). (**B**) Venn diagram of the number of genes with significant differential expression between *P. car* and *P. evo* legs before separation (orange) and after separation (blue) in parental species. (**C**) Principal component analysis of legs before and after separation in F1 hybrids. (**D**) Venn diagram of the number of genes with significant differential expression between *P. car* and *P. evo* alleles in legs before separation (orange) and after separation (blue) in F1 hybrids. Tissues include legs, bot, mid, top, pel, and tail.

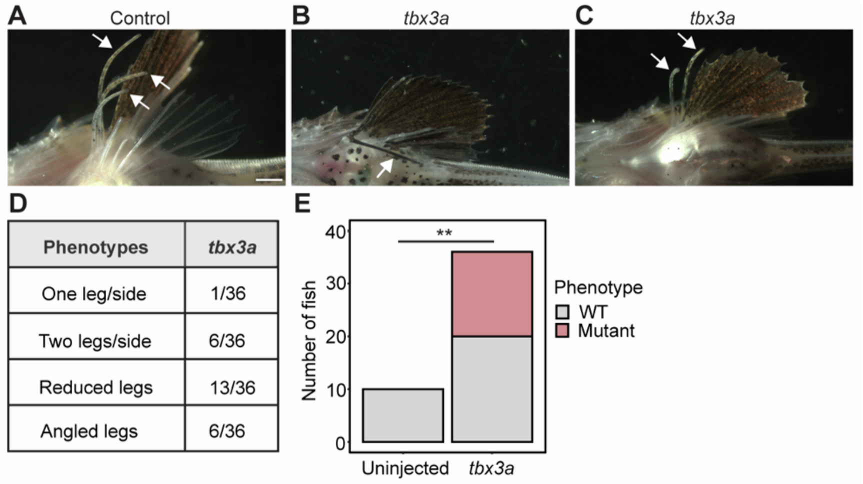

Figure S9. *P. evolans tbx3a* crispant phenotypes, related to Figure 3. (**A**) Control legs in a *P. evolans* juvenile. (**B**) An angled leg in a *tbx3a* crispant. (**C**) A *tbx3a* crispant with two legs. (**D**) Quantification of variable *tbx3a P. evolans* phenotypes. (**E**) Calculation of phenotype significance in *tbx3a* crispants. Fisher's exact test used for calculating significance. Exact *p-value:* $p = 0.009$. N = 10 controls and N = 36 *tbx3a* crispants. Scalebar, 1 mm.

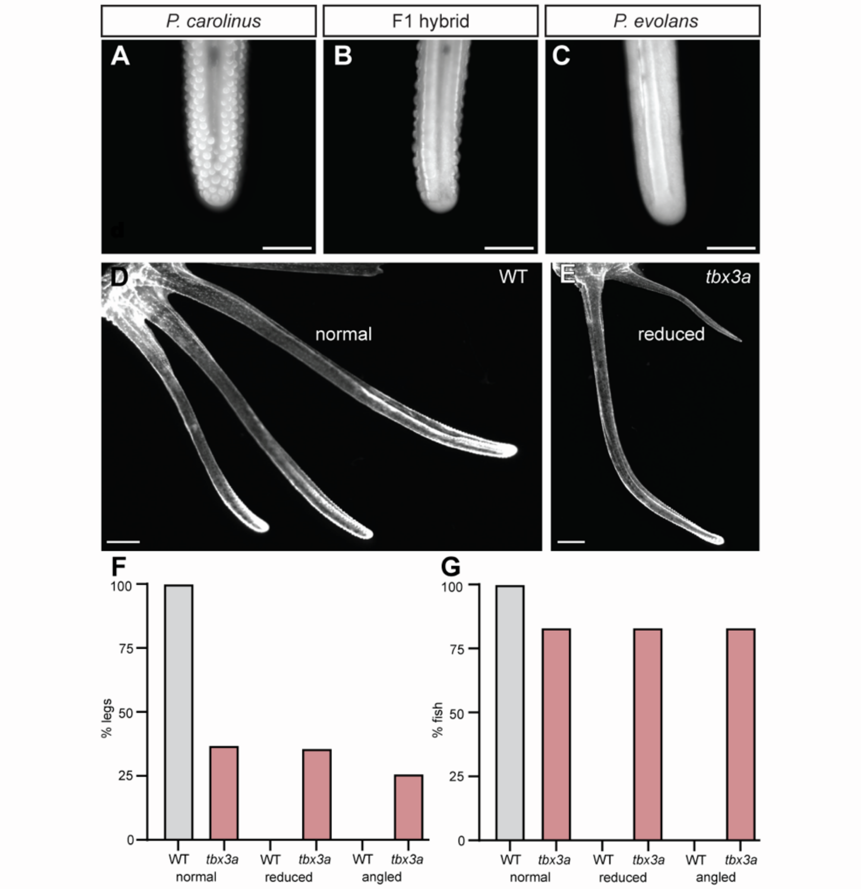

Figure S10. Papillae and leg phenotypes across species, hybrids, and *tbx3a* crispants, related to Figure 3. (**A**) A juvenile *P. carolinus* leg exhibits robust papillae. (**B**) An F1 hybrid shows intermediate papillae while a *P. evolans* animal lacks papillae (**C**). (**D**) Control *P. carolinus* animals have three normal legs while legs in *tbx3a* crispants can appear normal or reduced in size (**E**). (**F**) Quantification of different leg phenotypes in *P. carolinus* control and *tbx3a* crispants. (**G**) Quantification of the percentage of fish exhibiting phenotypes. N = 36 control legs and N = 35 *tbx3a* legs analyzed (**F**) and N = 6 control and N = 6 *tbx3a* crispant fish analyzed (**G**). Papillae/mm were measured in the same animals and quantified in Figure 3G. Scale bars, 500 µm (**A, B, C**); 1 mm (**D**, **E**).

Metric	Prionotus carolinus	Prionotus evolans
Total length	642,348,027 bp	819,076,282 bp
N50	18,828,545 bp	14,254,066 bp
L50	14	20
Largest scaffold	32,384,465 bp	35,408,593
Number of scaffolds	239	1,329
Number of gaps	243	827
Number of N's per 100 kbp	3.78	10.1
Complete and single-copy BUSCOs	98.04%	94.51%

Table S1. Haplotype resolved genome assembly metrics, related to Figure 1 and Figure 3.

Analysis Type:	PANTHER Overrepresentation Test (Released 20240226)				
Annotation Version and Release Date:	GO Ontology database DOI: 10.5281/zenodo.10536401 Released 2024-01-17				
Test Type:	FISHER				
Correction:	FDR				
**GO biological process complete**	**Reference gene list**	**Observed genes**	**Expected genes**	**Fold enrichment**	**FDR**
neuron differentiation (GO:0030182)	144	6	0.73	8.2	4.07E-02
generation of neurons (GO:0048699)	148	6	0.75	7.98	4.27E-02
cell surface receptor signaling pathway (GO:0007166)	199	8	1.01	7.91	7.17E-03
cell differentiation (GO:0030154)	402	9	2.04	4.41	4.88E-02
cellular developmental process (GO:0048869)	403	9	2.05	4.4	4.56E-02
system development (GO:0048731)	496	11	2.52	4.37	1.13E-02
multicellular organism development (GO:0007275)	592	12	3.01	3.99	1.12E-02
multicellular organismal process (GO:0032501)	699	13	3.55	3.66	1.24E-02
developmental process (GO:0032502)	808	15	4.1	3.65	5.60E-03
anatomical structure development (GO:0048856)	766	14	3.89	3.6	1.05E-02
regulation of cellular process (GO:0050794)	1580	18	8.03	2.24	4.00E-02
biological regulation (GO:0065007)	1748	19	8.88	2.14	4.22E-02

Table S2. Summary of GO biological process terms, related to Figure 1.

**AA position (Gasterosteus; Prionotus carolinus)**	**Conserved in Gasterosteus, Medaka, Zebrafish**	**Derived in Sea robins**
77	S (polar)	P (non-polar)
320	G (non-polar)	--
333 ; 332	K (+ charge)	N (polar)
334 ; 333	C (non-polar)	F (non-polar)
335 ; 334	F (non-polar)	I (non-polar)
354 ; 353	F (non-polar)	S (polar)
356 ; 355	D (- charge)	Y (polar)
643 ; 641	G (non-polar)	A (non-polar)
644 ; 642	T (polar)	A (non-polar)
707 ; 711	A (non-polar)	P (non-polar)

Table S3. Sea robin specific amino acid changes, related to Figure 3.

Supplemental References:

S1. Crowley, J.J., Zhabotynsky, V., Sun, W., Huang, S., Pakatci, I.K., Kim, Y., Wang, J.R., Morgan, A.P., Calaway, J.D., Aylor, D.L., et al. (2015). Analyses of allele-specific gene expression in highly divergent mouse crosses identifies pervasive allelic imbalance. Nature Genetics *47*, 353–360. https://doi.org/10.1038/NG.3222.

S2. Hu, C.K., York, R.A., Metz, H.C., Bedford, N.L., Fraser, H.B., and Hoekstra, H.E. (2022). cis-Regulatory changes in locomotor genes are associated with the evolution of burrowing behavior. Cell Reports *38*. https://doi.org/10.1016/j.celrep.2022.110360.
